# Few basepairing-independent motifs in the apical half of the avian HBV ε RNA stem-loop determine site-specific initiation of protein-priming

**DOI:** 10.1038/s41598-017-07657-z

**Published:** 2017-08-02

**Authors:** Markus Gajer, Katharina Dörnbrack, Christine Rösler, Bernadette Schmid, Jürgen Beck, Michael Nassal

**Affiliations:** University Hospital Freiburg, Department of Internal Medicine II/Molecular Biology, Hugstetter Str. 55, D-79106 Freiburg, Germany

## Abstract

Hepadnaviruses, including human hepatitis B virus (HBV), replicate their tiny DNA genomes by protein-primed reverse transcription of a pregenomic (pg) RNA. Replication initiation as well as pgRNA encapsidation depend on the interaction of the viral polymerase, P protein, with the ε RNA element, featuring a lower and an upper stem, a central bulge, and an apical loop. The bulge, somehow assisted by the loop, acts as template for a P protein-linked DNA oligo that primes full-length minus-strand DNA synthesis. Phylogenetic conservation and earlier mutational studies suggested the highly based-paired ε structure as crucial for productive interaction with P protein. Using the tractable duck HBV (DHBV) model we here interrogated the entire apical DHBV ε (Dε) half for sequence- and structure-dependent determinants of *in vitro* priming activity, replication, and, in part, *in vivo* infectivity. This revealed single-strandedness of the bulge, a following G residue plus the loop subsequence GUUGU as the few key determinants for priming and initiation site selection; unexpectedly, they functioned independently of a specific structure context. These data provide new mechanistic insights into avihepadnaviral replication initiation, and they imply a new concept towards a feasible *in vitro* priming system for human HBV.

## Introduction

**Figure 1:**
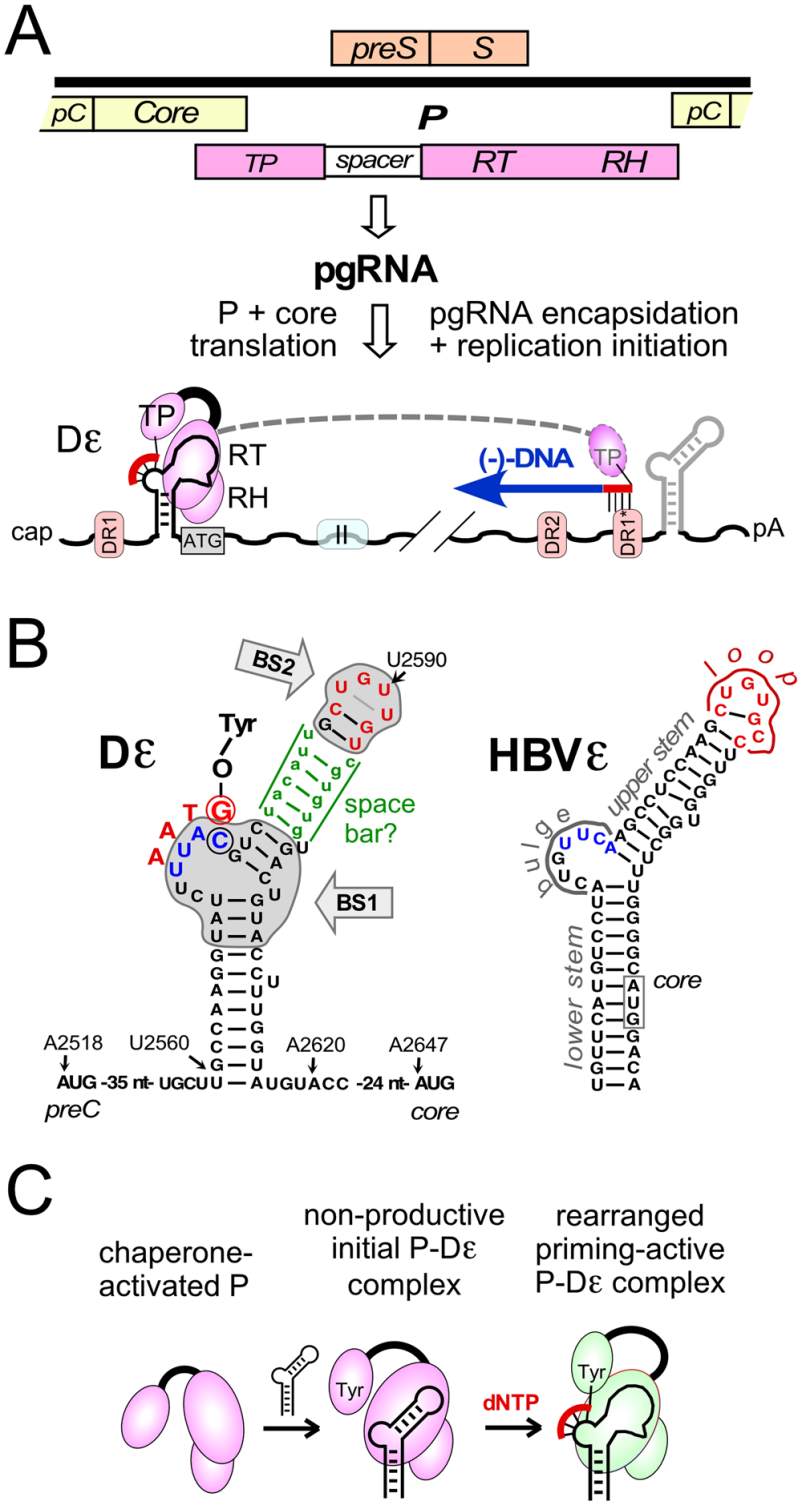
Key roles of the ε RNA stem-loop in hepadnaviral replication. (**A**) Protein-priming and minus-strand DNA synthesis. The 3 kb DHBV DNA genome is schematically shown on the top in linearized form; ORFs for preC (pC), core, preS/S and P with its Terminal Protein (TP), Reverse Transcriptase (RT) and RNase H (RH) domains are indicated. Pregenomic (pg) RNA, transcribed from transfected vector or, in infection, from covalently closed circular (ccc) DNA^43^, serves as mRNA for core and P protein, and as precursor for new RC-DNA. Binding of P protein to 5′ ε triggers co-encapsidation of the complex (in DHBV requiring additional downstream sequences symbolized by the box labeled “II”^7, 8^) into newly forming nucleocapsids (not shown) and synthesis of a short, TP-linked DNA oligonucleotide (“protein priming”); transfer to a sequence-matching acceptor at DR1* mediates minus-strand DNA synthesis. The subsequent steps towards RC-DNA are not shown. (**B**) Known functional subelements in Dε and comparison to HBV ε. The central bulge and the apical loop are established determinants for ε function. The initiation site is indicated by the encircled C at the 3′ end of the bulge, templating a G as first residue linked to TP. The connecting upper stem (green) might act as a space bar ensuring a specific distance between bulge and loop for optimal interaction with the assumed binding sites (BS1, BS2) on P protein^22^. HBV ε exerts a similar structure, except the upper stem structure is more stably basepaired. A general assignment for the ε subelements lower and upper stem, bulge and loop is given on the HBV ε structure. The apical sequences in Dε and ε given in red denote the classical heptaloop (DHBV) and hexaloop (HBV), the intra-loop pairings shown are derived by NMR. (**C**) Distinct steps during protein-priming deduced from *in vitro* priming systems available for DHBV but not HBV. P protein activation by chaperones (dispensable for miniDP used here) enables initial binding to Dε; mutations in Dε can cause an arrest at this stage. Rearrangements in RNA and protein provide the complex with priming activity (green protein color), enabling synthesis of the bulge-templated DNA oligo (red).

Hepadnaviruses, with the important human pathogen hepatitis B virus (HBV) as their prototype, are small enveloped DNA viruses that replicate via protein-primed reverse transcription of a pregenomic (pg) RNA intermediate^1, 2^ (Fig. 1A). A crucial cis-element for replication initiation is a highly conserved bipartite RNA hairpin, the encapsidation signal ε, which comprises a lower and an upper stem, a central bulge and an apical loop (Fig. 1B). ε is specifically recognized and bound by the viral polymerase, P protein. Productive ribonucleoprotein (RNP) complex formation is accompanied by structural rearrangements (Fig. 1C) in protein and RNA^3–6^, and triggers co-packaging of pgRNA and P protein into newly forming nucleocapsids, i.e. encapsidation (in DHBV facilitated by the downstream “region II”)^7, 8^, yet also the synthesis of a 3–4 nucleotide (nt) DNA oligo that is templated by the 3′ half of the ε bulge; its first nt is covalently linked to a Tyr-residue in P protein’s Terminal Protein (TP) domain (protein-priming). For extension into full-length minus-strand DNA the oligo is translocated to a complementary acceptor at the 3′ proximal direct repeat DR1* (Fig. 1A). At least the two 3′ terminal nt of the DNA oligo must match the acceptor site for translocation to this site^9, 10^, therefore only oligos initiated at the correct position in the ε bulge ensure formation of proper minus-strand DNA and eventually the hepadnavirus-typical relaxed circular (RC) DNA genome present in mature nucleocapsids and enveloped virions^1, 2^.

Hence ε RNA contains elements that activate P protein, similar to the RNA components in telomerase^11^ and in the CRISPR/Cas9 system^12^, and must also encode the information for proper positioning of P protein over the initiation site at the 3′ end of the bulge; in most conventional DNA copying systems the initiation site is defined by the extendable 3′ end of a nucleic acid primer, e.g. a host tRNA in retroviruses. Current knowledge on how this is achieved in hepadnaviral P - ε complexes is limited. To date it has been impossible to isolate functional RNPs in sufficient quantity and quality for direct structural investigation. For HBV, mutational analyses were largely confined to using pgRNA packaging and viral DNA formation in transfected cells as readout. *In vitro* HBV RNP complex formation has been achieved but without detectable DNA synthesis activity^13^; we refer to this a non-productive binding. The most advanced cell-free HBV priming system relies on co-expression of tagged P protein and ε RNA in mammalian cells, with subsequent affinity purification^14, 15^; however, the RNA in the isolated RNPs cannot be exchanged. Hence for functional studies using P protein and/or ε RNA mutants each new RNP must individually be generated, precluding larger scale analyses. For DHBV, in contrast, *in vitro* priming systems are long established^16^. Priming activity of recombinant full-length or near full-length P protein requires cellular chaperones, as present in rabbit reticulocyte lysate^6, 17–19^ or added in substance^18^, in particular heat shock proteins Hsc70 and Hsp40^4, 5, 20^. This chaperone dependence is overcome by terminal truncations in P protein^20, 21^, with the respective “miniDP” proteins providing the most feasible systems to investigate Dε-dependent protein-priming. Conversely, the impact of ε mutations can even be analyzed in the context of genuine DHBV infection of ducks^22^.

Collectively, the available data indicate that both the bulge and the loop are important for a productive interaction with P protein. Bulges, loops and unpaired residues within an RNA double-helix offer a wealth of unique recognition surfaces and thus are predestined as highly specific binding sites, including for P protein. The actual presence of both elements in free HBV and DHBV ε RNA was biochemically confirmed^6, 23–25^, and NMR investigations revealed further details, including non-canonical basepairs, in the previously assumed (“classical”) hepta- and hexaloop sequences capping the upper stem, creating a tetraloop in Dε^26^ and a pseudotriloop in HBV ε^27^.

By necessity, bulges and loops are defined by their flanking double-helical regions. In ε the stable lower stem determines the start of the bulge, and the upper stem defines its end and closes the apical loop. Furthermore, the functional need for both bulge and loop implies their concerted interaction with P protein, either directly or through a separate loop-binding factor^28^; hence the connecting upper stem could also act as a molecular ruler that determines the spacial distance and orientation of the two subelements, optimally positioning them for productive binding to P protein, including for proper initiation site selection (Fig. 1B).

Phylogenetic conservation of the bipartite ε structure amongst different hepadnaviruses and early mutagenesis studies^23, 24, 28, 29^ supported such a function for the upper stem. Yet, in priming-competent Dε complexes this region is opened up^6^, and various *in vitro* selected Dε variants lacking basepairing potential in the top part of the upper stem supported *in vitro* priming^30^ and even chronic infection in ducks^22^.

However, all previous studies addressed only small sections of the upper ε stem, leaving most of the natural sequence and, given the dependence of RNA structure on sequence, structural context intact. Hence which sequence- and/or structure-dependent determinants enable ε RNA to activate P protein and present a specific nt for replication initiation remained open.

We here sought to comprehensively interrogate the entire apical Dε part for determinants that allow for a productive interaction with P protein, using as readout *in vitro* priming, replication in transfected cells and, for selected variants, infectivity in ducks. Altogether, an unexpectedly large number of length, sequence and structure modifications had only modest impacts on Dε function, including selection of the authentic initiation site. Forward selection using a replication-dependent systematic evolution of ligands by exponential enrichment (SELEX) procedure revealed a subsequence of the classical heptaloop sequence, GUUGU, and a single G residue immediately following the bulge as the only crucial determinants in the apical Dε part, including for selecting the genuine 3′ terminal bulge nt (bulge position 6) as initiation site; again, however, no need for a specific structure context became obvious. We then confirmed our suspicion that the bulge region itself harbors functionally important determinants for initiation site selection via combinatorial mutations targeting bulge position 6 and the unpaired U residue opposite the bulge. Altogether, the results suggested a revised model for a productive DHBV P protein - Dε interaction whereby the critical determinants in the RNA are a single-stranded bulge region (guaranteed by the stable lower stem), a G residue following the bulge 6 position with an oppositely located pyrimidine, plus a GUUGU motif in not too far a distance but independent from a specific upper stem structure. This was verified by the priming activity and replication-competence of artificial Dε RNAs in which these primordial elements were embedded into a completely unstructured context. Hence accessibility of a few crucial RNA determinants is the main prerequisite for a functional upper stem sequence.

These results shed new mechanistic light on replication initiation in avian hepadnaviruses and have also implications for mammalian HBVs, including a new concept as to why human HBV ε with its highly stable upper stem structure does not support *in vitro* priming. The methods developed in this study should provide suitable tools to test this concept.

## Results

### The exact bulge-loop distance is not decisive for priming activity and initiation site selection

**Figure 2:**
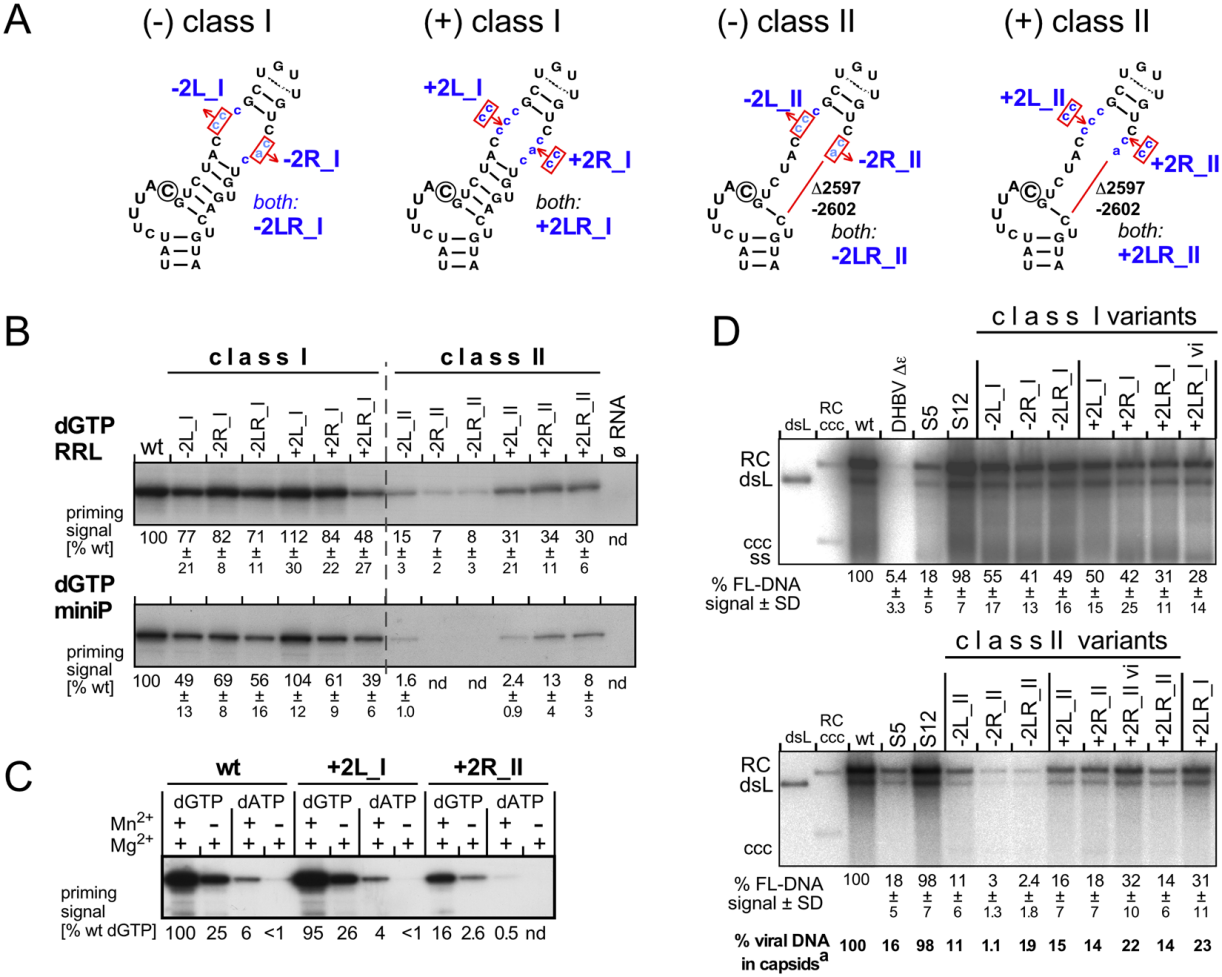
Interrogating a potential space-bar function of the upper Dε stem. (**A**) Sequence alterations in class I and class II variants. The distance between bulge and loop in the replication-proficient Dε variant S12^22^ was altered by deleting two of its non-authentic residues on the left or right upper half-stem or both, or by analogously introducing two extra C residues on one or both sides (class I). The same changes were introduced in a variant lacking *a priori* six nt in the lower right half-stem (class II). The encircled C represents the dominant initiation site. (**B**) *In vitro* priming activities. The indicated Dε variants were *in vitro* transcribed and subjected to α^32^P-dGTP priming assays using full-length DHBV polymerase in rabbit reticulocyte lysate (RRL), or recombinant miniDP protein. ^32^P-labeled P protein was visualized, after SDS-PAGE, by autoradiography. Signal intensities were quantified by phosphorimaging; numbers below each lane show the mean relative priming signals ± standard deviation (SD; n ≥ 3) compared to wt Dε RNA which was set to 100%; nd, not detectable. Analogous data for α^32^P-dATP are shown in Supplementary Fig. S1. (**C**) No impact of typical class I and class II mutations on dNTP specificity during *in vitro* priming. MiniDP priming assays were performed using either α^32^P-dGTP or α^32^P-dATP in the presence of only Mg^2+^, or Mg^2+^ plus Mn^2+^. In either constellation, dGTP was incorporated ~20-fold more efficiently than dATP. (**D**) Class I and class II variants support viral replication. LMH cells were transfected with pCD16 vectors bearing the indicated mutant sequences in both 5′ and 3′ Dε. Vectors encoding wt-DHBV16 (wt), a variant defective in 5′ Dε (DHBV Δε), and the upper stem variants S5 and S12^22, 30^ served as controls. DNA from cytoplasmic nucleocapsids was analyzed by Southern blotting using a^32^P-labeled DHBV DNA probe. Numbers indicate mean signal intensities ± SD (n ≥ 3) of full-length DNAs (RC + dsL) relative to wt-DHBV which was set to 100%.

In the largely double-helical wild-type Dε the distance between bulge and loop is around 5–6 nm^26^. To modulate this distance with minimal induction of new, alternative basepairings we used as parent a Dε variant, S12^30^, in which the top part of the upper stem lacks basepairing potential; however, the five basepairs on top of the bulge and the two basepairs closing the tetraloop can form (Fig. 2A), as in heron HBV (HHBV) ε^26^. S12 is priming-active *in vitro*, and in the context of the DHBV genome, supports replication in transfected LMH cells as well as in ducks *in vivo* ^22^.

In a first set of variants (termed “class I”) we deleted two residues from the left or the right half-stem (−2L_I; −2R_I), or both (−2LR_I); alternatively, two C residues each were inserted in the left or right half-stem (+2L_I; +2R_I), or both (+2LR_I). An analogous series of more severely altered variants (class II) were based on a derivative in which 6 nt at the base of the right half-stem (positions 2597–2602) were *a priori* deleted (mutants −2L_II and so on). Notably, variant −2LR_II lacks 10 of the totally 27 nt in the Dε upper stem.

We then assessed the ability of the variant RNAs to support *in vitro* priming with α^32^P-labeled dGTP, the natural first nt of minus-strand DNA templated by the 3′ terminal C of the bulge (b6 position), either with full-length DHBV P protein *in vitro* translated in rabbit reticulocyte lysate (RRL) or using a truncated recombinant P protein variant (miniDP) that retains priming activity without any protein co-factors^31^. Reactions with wt Dε or without RNA served as controls. Reaction products were separated by SDS-PAGE and band intensities of ^32^P-labeled P protein were quantitated by phosphorimaging (Fig. 2B, *top panel*). All class I mutants produced similarly strong signals as wt Dε in either system, except for a slightly stronger reduction with the most extended variant +2LR_I. Hence adding or deleting up to 4 nt from the upper stem had little effect on priming efficiency with dGTP.

In contrast, only very weak signals (though still higher than in the no-RNA control) were obtained with the class II RNAs lacking additional two or four nt, whereas adding back two or four nt partially rescued higher priming activity (Fig. 2B, *bottom panel*). To test whether the reduced class II priming signals reflected a general reduction in priming efficiency or an altered dNTP preference caused by a shift in initiation site we repeated all experiments using α^32^P-dATP (Supplementary Fig. S1) which might be templated by any of the various U residues around the genuine C template (Fig. 2A). In line with previous results^31^, the dATP signals with wt Dε were much weaker than with dGTP, and the same held for all mutants. Even inter-mutant variations were highly similar to those with dGTP. We also assessed whether Mn^2+^, routinely present in our miniDP assays because it stimulates priming^31^, affects dNTP preference. To this end, variants +2L_I and +2R_II were subjected, alongside wt Dε, to miniDP priming assays with dGTP or dATP, and in the presence of only Mg^2+^, or Mg^2+^ plus Mn^2+^ (Fig. 2C). For all RNAs, Mn^2+^ stimulated dGTP (by four- to fivefold) and also dATP utilization; however, the priming signals with dATP remained ten- to twenty-fold lower than with dGTP, corroborating a comparably strong dGTP preference of the variants as seen with wt Dε and indicating that a C residue acted as template.

To investigate whether the length-modified upper stems in the Dε variants affected replication as a whole, we transferred the variant sequences (plus two *in vivo* derived derivatives of +2LR_I and +2R_II, labeled with the suffix “vi”; see below) into the DHBV expression vector pCD16. Upon transfection into LMH cells all constructs produced similar amounts of cytoplasmic capsids (Supplementary Fig. S1). Southern blotting of the isolated capsid-borne viral DNAs revealed two- to three-fold lower DNA levels for the class I variants (Fig. 2D, *top panel*), and stronger reductions for the class II variants (Fig. 2D, *bottom panel*) compared to wt DHBV; this was confirmed by direct detection of the viral DNAs in intact capsids (Supplementary Fig. S1). Altogether, the extent of DNA signal reduction for each variant paralled that seen in the *in vitro* priming assays. Most notably, the patterns of replicative DNA intermediates (RC, relaxed circular; dsL, double-strand linear) were indistinguishable between variants and wt DHBV. Hence also in the cellular setting were all variants able to generate oligo primers supporting formation of full-length viral DNA, in line with their using the authentic priming initiation site; this was corroborated by mapping the minus-strand DNA 5′ ends by primer extension (Supplementary Fig. S1), where the major signals from all variants comigrated with those from wt DHBV and their relative intensities matched those seen in the previous assays. Hence minor changes in the distance between Dε bulge and apical loop (class I mutations) were well tolerated and major changes (class II mutations) affected the efficiency but not the accuracy of initiation site selection during priming.

We also assessed *in vivo* infectivity of selected variants (−2R_I, −2LR_I, +2LR_I; all class II mutants except +2L_II). All variants except −2R_II and −2LR_II with the poorest *in vitro* performance were able to establish viremia, although with later onset and substantially lower maximal titers than the wt DHBV controls (Supplementary Fig. S2). Sequencing revealed no difference to the inocula for variants −2R_I, −2LR_I and −2L_II; however, over time variant +2LR_I lost one of the two extra C residues in the left half-stem, in variant +2R_II one of the two extra C residues in right half-stem was mutated to A (Supplementary Fig. S2). In transfected cells, the latter variant (termed +2R_II vi), but not the former variant (termed +2LR_I vi), showed an increased replication capacity (Fig. 2D), possibly indicating a positive selection *in vivo*.

Altogether, these experiments confirmed that substantial alterations in the length and sequence of the Dε upper stem do not fundamentally compromise Dε function as origin of replication or as encapsidation signal. In particular, for most variants initiation site selection remained sufficiently accurate to enable formation of fully functional genomes over many generations.

### In-cell SELEX as a means to identify functional determinants in Dε

Given the modest impact of the upper stem modifications seen above we next focussed on the Dε subelements that were left intact in the class I and class II mutants, i.e. the loop and the bulge with their immediate vicinity.

To interrogate a larger sequence space we employed a similar SELEX approach as before^30^, however using as selection principle replication competence of complete viral genomes in cells rather than *in vitro* binding to P protein of short Dε RNAs. Only Dε sequences competent for pgRNA packaging, priming and reverse transcription should yield progeny RC-DNA. We further reasoned that a requirement for defined sequences in a subregion of Dε would result in the selection of one or few individual sequences whereas the absence of such a selection would indicate the absence of specific sequence requirements.

To this end, we transfected DHBV expression vector pools randomized at the desired Dε regions (see Materials and Methods, and Supplementary Methods) into LMH cells, then enriched viral particles and used nuclease-resistant DNA as template for another subgenomic PCR, the products of which served to create a new vector pool. This procedure was repeated several times. For characterization, we used pool sequencing combined with cloning of individual sequences, various of which were also analyzed for replication competence. As targets for randomization we chose the bulge, the loop, and the seven nt of the upper left stem (ULS) immediately following the bulge.

### In-cell SELEX rapidly selects for the authentic template sequence

**Figure 3:**
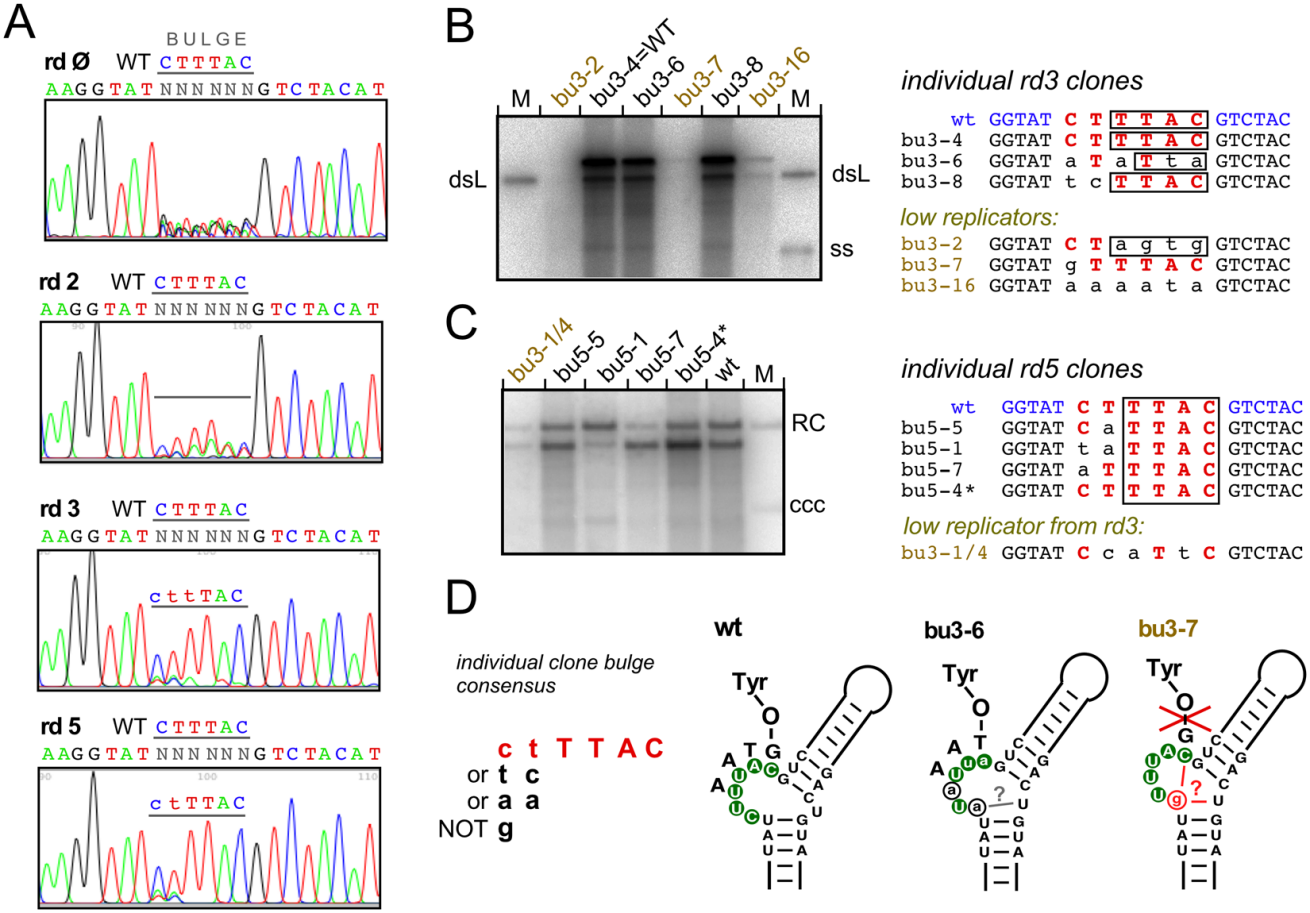
In-cell SELEX efficiently selects for wt-like Dε bulge template sequences. A pool of pCD16-Δ3′ε vectors bearing random nt at the six bulge positions was subjected to sequential transfection/selection rounds as detailed in Supplementary Methods. (**A**) Pool sequencing. Sequencing chromatograms were obtained from the recombinant vector pool before transfection (round Ø) and the new vector pools generated after the indicated number of SELEX rounds. Note the increasing proportion of sequences conforming to the genuine template sequence TTAC, and the persistence of some heterogeneity at bulge positions 1 and 2. (**B**) Replication capacity of individual round 3 clones in transfected LMH cells. The round 3 pool comprised a mixture of high and low replicators; their sequences are shown on the right; the bulge sequence is in red, the template region is boxed. Lower case black letters indicate non-wt nt. (**C**) Replication capacity of individual round 5 clones. All sampled individual clones were replication-competent; round 3 clone bu3-1/4 served as low replicator control. (**D**) Functional bulge consensus sequences derived from individually characterized clones. Despite some heterogeneity at bulge positions 1 and 2, most high replicators contained the authentic template sequence TTAC, low replicators deviated from that sequence (e.g. bu3-2, bu3-16). The schemes show wt-Dε (SELEX-targeted nt in white letters with green background) vs. the exceptional clones bu3-6 and bu3-7. Clone bu3-6 replicated at near wt-level despite an altered template sequence; non-wt nt are given in lower case. However, it may specify a primer similar to the wild-type primer GTAA. Clone bu3-7 replicated poorly despite only a single C > G at bulge position 1 (in red), possibly due to an altered bulge structure as indicated.

Given the importance of a properly ε-templated oligo primer for the subsequent replication steps, randomizing the bulge allowed to validate the procedure. Figure 3A shows the relevant parts of the pool sequence chromatograms over five selection rounds. As expected, sequences conforming to the wt bulge sequence 5′ ctTTAC (template region in capitals) were rapidly enriched while some heterogeneity persisted at the two 5′ terminal positions. This was confirmed by individual clone sequences obtained after 3 and 5 selection rounds and their replication capacities when singly transfected into LMH cells (Fig. 3B,C). The proportion of poorly or not at all replicating clones decreased with each new selection round (see Supplementary Fig. S3 for additional individual clones), and replication-competence correlated strongly with the presence of the authentic template sequence TTAC. Weak replicators after round 3 (Figs 3B and S3) contained severely altered template regions (bu3-2, bu3-16). A seeming counter-example was clone bu3-7 with a single C > G exchange at bulge position 1; however, this exchange could engage the initiating C and/or the unpaired U opposite the bulge into new basepairs (Fig. 3D; see also below). Conversely, clone bu3-6 had only two of the six authentic bulge positions preserved but replicated well. Notably, this bulge sequence may be regarded as a deletion of just the first template nt, specifying a wt-similar primer that still carries the TAA motif matching the DR1* acceptor site. While these interpretations will have to be confirmed by subsequent experiments, the results as such corroborated the viability of the approach.

### In-cell SELEX identifies a critical subsequence in the loop

**Figure 4:**
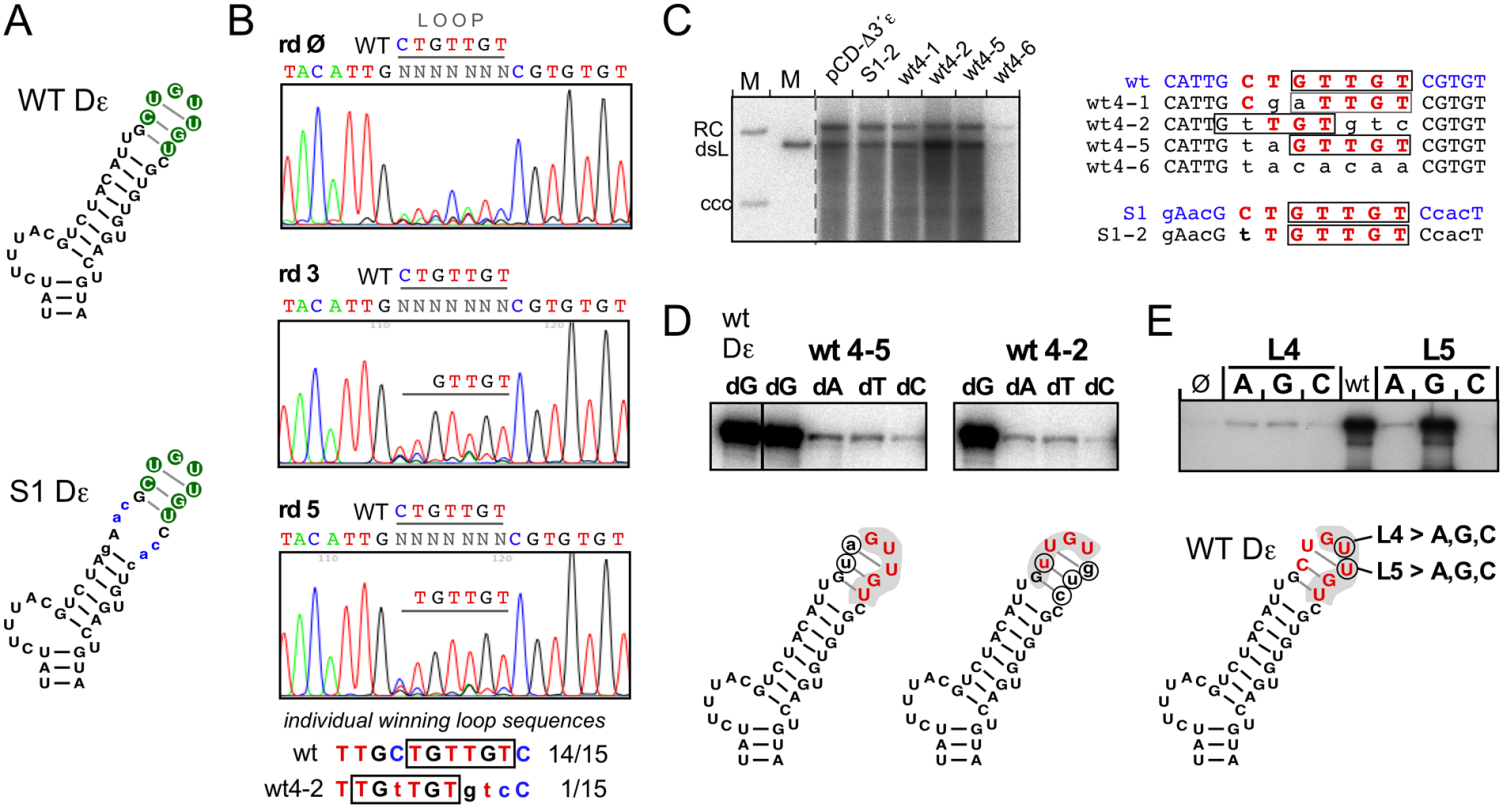
In-cell SELEX reveals the importance of the GUUGU motif in the apical loop of wt-Dε. SELEX experiments were performed using a starting pool carrying a randomized classical loop sequence in the context of wt-Dε. (**A**) Positions of the randomized loop sequences in wt-Dε and variant S1. Targeted nt are shown as white letters with green background. (**B**) Pool sequencing chromatograms. Note the rapid emergence of the GUUGU motif already after round 3. After round 5, 14 of 15 individual clones carried the wt-loop sequence, one (wt4-2; already seen after round 4) still contained the TGTTGT motif though a shifted position, as shown at the bottom. (**C**) Replication capacity of individual round 4 sequences. Clone wt4-6 with a completely non-wt loop sequence replicated poorly; all others replicated at near wt-levels, including clone S1–2 derived from the next SELEX experiment. Note the common presence of the GUUGU motif (aUUGU in clone wt4-1) though at a shifted position in clone wt4-2. (**D**) No impact of GUUGU-adjacent residues on dNTP specificity during *in vitro* priming. Variants wt4-5 (non-wt nt at loop positions 1 and 2) and wt4-2 (shifted GUUGU motif) were analyzed by miniDP *in vitro* priming assays with either dGTP, dATP, dTTP or dCTP as only dNTP. Both exerted a strong preference for the authentic dG. (**E**) Strong impact of mutations within the GUUGU motif on priming-activity. The central U residues at loop positions L4 (U2590) and L5 (U2591) were individually mutated as indicated, then analyzed by miniDP priming assays with dGTP.

Next we applied the procedure to the classical loop sequence CUGUUGU^23^, in the context of either wt Dε or the previously described viable variant S1^22, 30^ in which the sequence underlying the loop cannot basepair (Fig. 4A). As shown in Fig. 4B for the wt Dε based pool, there was a rapid selection of sequences carrying the authentic UGUUGU motif, except selection of the leading U residue was slower; the highest level of heterogeneity persisted at loop position 1. Of 15 individual clones from round 5, 14 carried the wt loop sequence, one corresponded to the round 4 clone wt4-2 which harbors an upstream-shifted UGUUGU motif (Fig. 4B,C).

The replication phenotypes of several individual round 4 clones diverging from the authentic loop sequence are shown in Fig. 4C. Clone wt4-6 with no match to the authentic loop sequence replicated only marginally, whereas clone wt4-5 with mutations at loop positions 1 and 2 nt and clone wt4-2 with an upstream shifted GUUGU motif replicated as well as the wt DHBV control vector. The same held for clone wt4-1 where the leading G was replaced by A; however, the low abundance of A residues at this position suggests a clear preference for G. Hence the GUUGU motif appeared crucial for Dε function. In miniDP priming assays using either of the four dNTPs clones wt4-2 and wt4-5 both retained a wt-like preference for dGTP (Fig. 4D). To confirm the importance of the GUUGU motif as such we replaced the two central U residues (loop positions L4 and L5) individually by all other nt. At L4, all non-U nt caused a drastic drop in dGTP priming efficiency, at L5 a G was tolerated but A and especially C gave only extremely weak signals (Fig. 4E).

**Figure 5:**
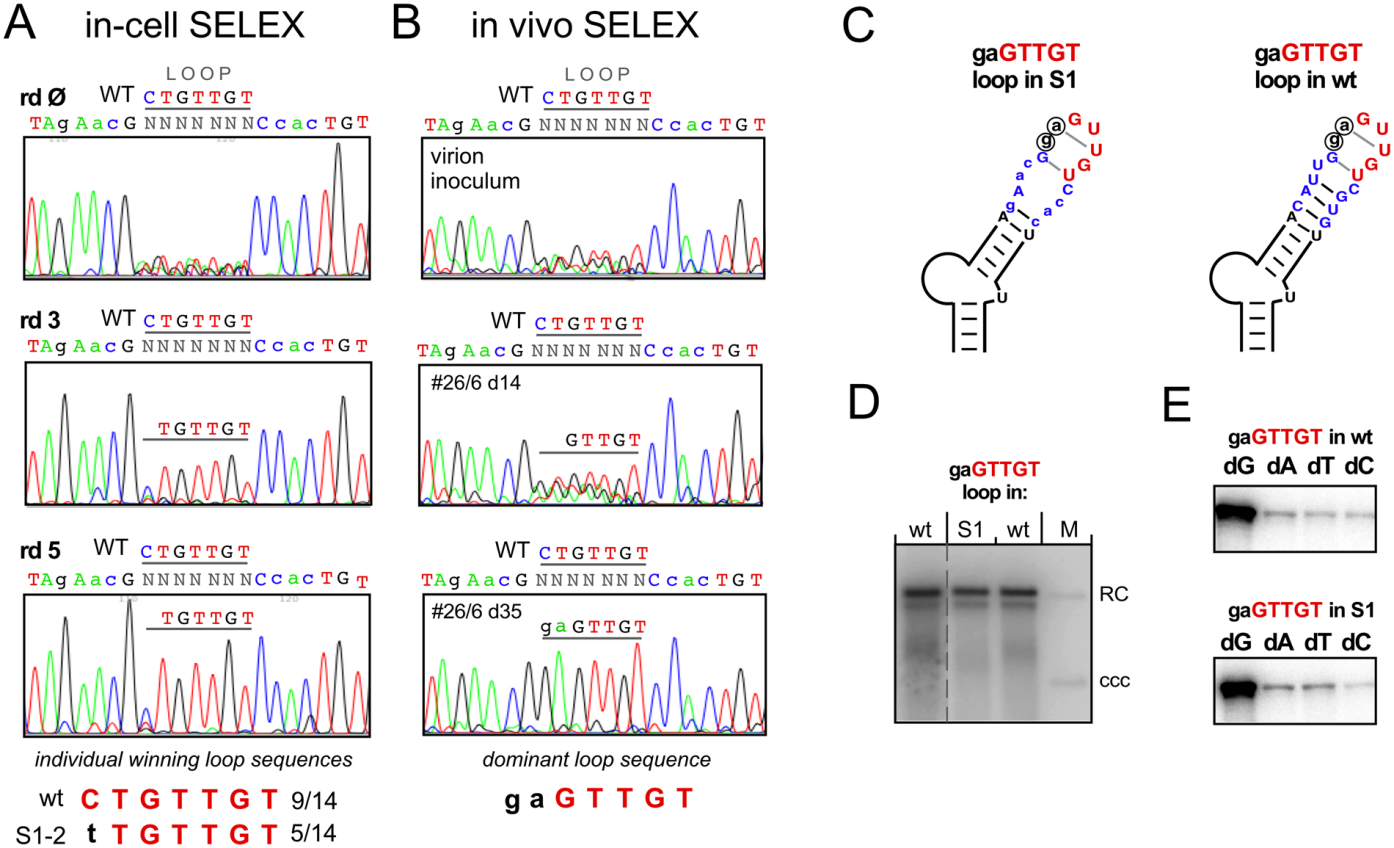
In-cell SELEX and *in vivo* selection confirm context-independence of the GUUGU motif. SELEX experiments were performed using a starting pool carrying a randomized loop sequence in the context of variant S1 (see Fig. 4A). (**A**) In-cell SELEX pool sequencing chromatograms. As in wt-Dε context sequences carrying the UGUUGU motif were rapidly selected, with some heterogeneity at loop position 1. In the sequence assignments above each chromatogram the S1-specific nt exchanges are indicated by lower case lettering. The winning sequences after round 5 are shown at the bottom. (**B**) Selection of the GUUGU motif *in vivo*. Two ducklings were inoculated with transfection-derived virions from the round 1 pool. Sequencing chromatograms are derived from the inoculum and serum samples of DHBV-positive animal #26/6 collected on d14 and d35 DNA post inoculation which revealed gaGTTGT as dominant winning loop sequence. (**C**–**E**) The non-wt gaGUUGU motif is functional in S1 and wt-Dε context. (**C**) Schematic representation of the modified loop sequence in S1 and wt-Dε. (**D**) Wild-type like replication capacity in transfected LMH cells. (**E**) No impact on dNTP specificity during *in vitro* priming.

To address a potential impact of the loop flanking sequences we repeated the SELEX experiments in the context of variant S1 (see Fig. 4A). Again there was a rapid selection of the wild-type sequence at the six 3′ proximal loop positions, with persisting heterogeneity at position 1 (Fig. 5A). Of 14 individual clones isolated after round 5, nine conformed to the wild-type sequence, five had a T instead of C at position 1 (termed S1–2); this sequence also replicated well (Fig. 4C). Hence also in S1 context the GUUGU motif was strongly selected. To confirm the physiological relevance of these findings we finally inoculated two ducklings with virions from LMH cells transfected with the S1-based starting pool (Fig. 5B). One of the ducklings developed detectable viremia. Pool sequencing of a serum sample collected on day 14 post inoculation revealed a mixture of sequences, however the GUUGU motif was already prominently visible. The day 35 sample showed one dominant sequence, gaGUUGU (Fig. 5B). To address whether the leading ga dinucleotide reflected an adaptation to the S1 context (Fig. 5C), we compared the replication capacity of the gaGUUGU loop sequence within S1 vs. wt Dε context in transfected LMH cells, however without detecting substantial differences to genuine wt DHBV (Fig. 5D); also, both variants exerted a wt-like preference for dGTP as first nt in *in vitro* priming (Fig. 5E).

Together, these data strongly support the functional importance of the 3′ terminal GUUGU motif but not the first two positions in the classical Dε loop sequence. Moreover, both gaGUUGU variants cannot form the C-G pair (Fig. 5C) that stabilizes the tetraloop in wt Dε^26^. Hence the classical loop subsequence GUUGU appeared to largely represent a *linear* determinant for Dε function.

### In-cell SELEX defines a critical G residue following the initiation site

**Figure 6:**
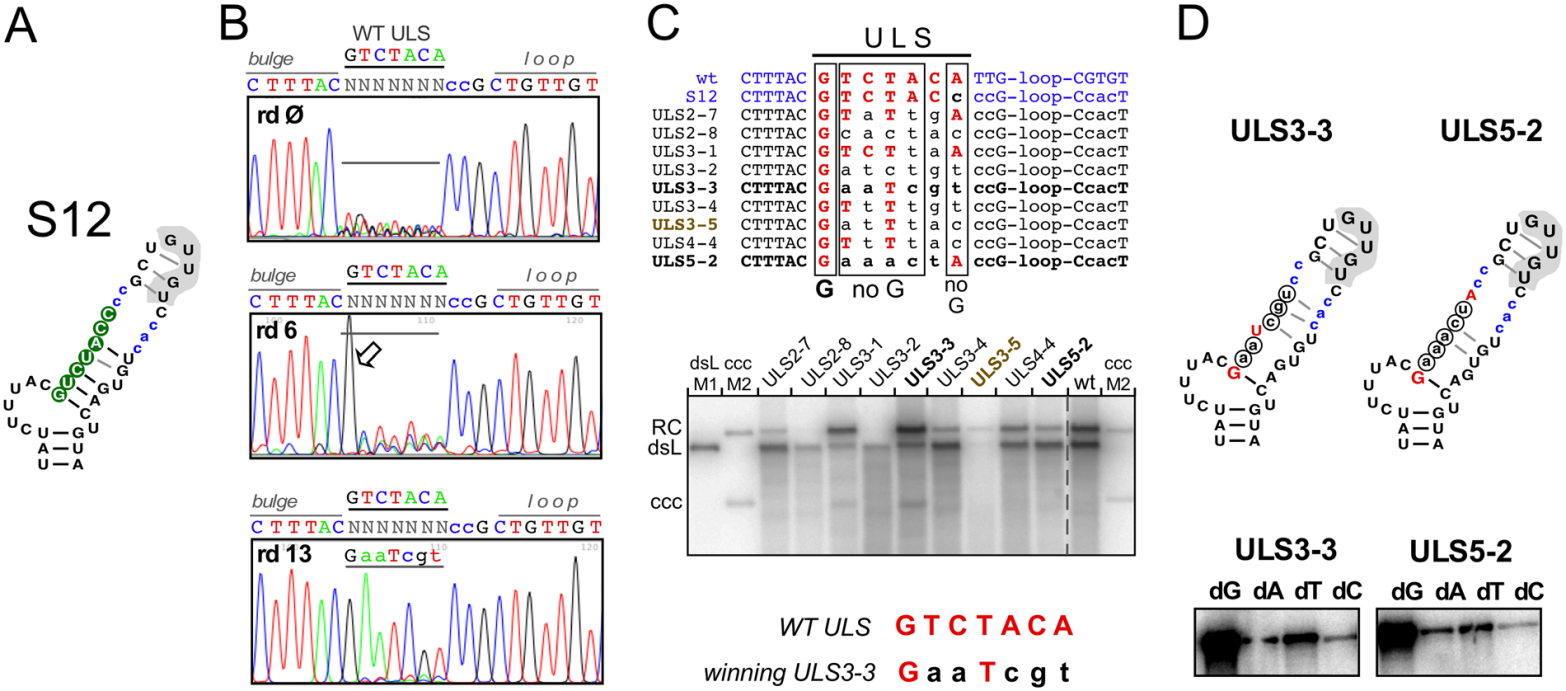
In-cell SELEX reveals the importance of a G residue following the initiation site. SELEX experiments were performed using a starting pool randomized at the seven 5′ proximal residues of the left upper stem (ULS) in the context of variant S12. (**A**) Schematic representation of Dε variant S12. Residues in lower case differ from wt-Dε; randomized nt are shown as white letters on green background. (**B**) In-cell SELEX pool sequencing. Except for a strong selection of G at ULS position 1 (arrowhead) the randomized region remained heterogenous after round 6, and a dominant sequence (ULS3-3) emerged only after round 13. (**C**) Replication capacity of individual ULS sequences. A collection of clones isolated after various SELEX rounds (*top panel*) were analyzed in parallel with wt-DHBV by Southern blotting after transfection into LMH cells. Even clones from early rounds universally contained a G at ULS position 1 but differed substantially from each other at the remaining six ULS positions. Differences in the ratio of RC-DNA vs. dsL-DNA signals as marked as here were not seen in all experiments. (**D**) No impact of multiple non-wild-type nt in the upper left half-stem on dNTP specificity. Wt nt selected during SELEX are shown in red upper case, non-wt nt in encircled lower case. Clones ULS3-3 (the winning sequence after 13 rounds) and ULS5-2 (with wild-type nt only at the first and last randomized ULS position) were analyzed by miniDP priming assays using a single dNTP; both exerted a strong preference for dGTP. A quantitative assessment of the replication performance of ULS3-3 vs. wt-DHBV in LMH cells is shown in Supplementary Fig. S4.

Finally we applied the SELEX procedure to the seven nt of upper left half-stem (ULS) immediately following the bulge. A requirement for basepairing should result in selection of nt that are complementary to the right half-stem, including for clarification whether the 3′ end of the bulge needs to be closed by a stable double-stranded structure (and thus would meet the definition of a bulge). Using as recipient variant S12^22, 30^ with an even more open top upper stem than in S1 allowed to interrogate the importance of basepairing in almost the entire upper stem sequence (Fig. 6A). Not the least, potential sequence-specific features in the connecting sequence between bulge and loop might be revealed.

Monitoring selection by sequencing the pools (Fig. 6B) plus individually isolated clones (Fig. 6C) after various selection rounds uncovered two striking features. First, even after six rounds no specific sequence emerged at positions 2 to 7 of the randomized ULS sequence; second, and much in contrast, position 1 exclusively contained a G residue, as in wt Dε. This was also true for all functional clones isolated from prior selection rounds (Fig. 6C), suggesting an important role for the specific nt following the template bulge but not the rest of the left upper half-stem. There, the only common feature was the absence of G residues at positions 2 to 5 and position 7 of the randomized sequence. Only after 13 selection rounds (though not yet after 11 rounds; see Supplementary Fig. S4) did a single winning sequence emerge that had previously been seen after round 3 as one (clone ULS3-3) of various different sequences and replicated well (Fig. 6C); in direct competition with wt-DHBV, ULS3-3 replicated about one third as efficiently (Supplementary Fig. S4). Notably, the only common sequence features in the randomized region of ULS3-3 vs. wt Dε were the G following the bulge, plus a U at position 4 (Fig. 6D), leaving nearly no basepairing potential in the entire upper stem; in particular, basepairing directly above the template bulge would be limited to a single G-C pair. In miniDP priming assays ULS3-3 as well as clone ULS5-2 with only the first and seventh of the randomized ULS positions occupied by wt-residues exerted a similarly strong preference for dGTP as wt Dε (Fig. 6D). Though based on a limited number of variants, the data do not provide any positive evidence for a major role in initiation site selection of the ULS sequence downstream of the leading G or its ability to pair with the right half-stem.

Altogether, the SELEX data revealed surprisingly few specificity determinants for a productive Dε - P protein interaction in the entire upper stem, except a G residue following the initiation site and a downstream GUUGU motif, functional in various sequence and structure contexts. Thus a remaining option for proper initiation site selection was that selective recognition of the template region relies largely on the bulge region as such, including its immediate vicinity.

### The unique architecture of the bulge region is important for efficient priming and initiation site selection

**Figure 7:**
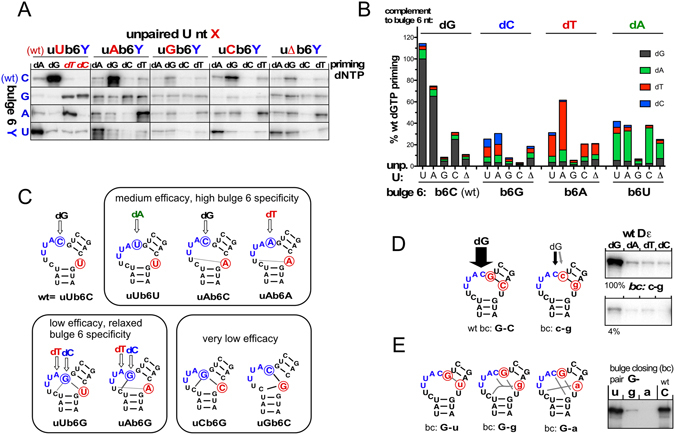
Impact of bulge region architecture on initiation site selection. Dε RNAs in which the unpaired U opposite the bulge (uA, uG, uC, or uΔ where the U was deleted) and the initiation site C at the b6 position (b6G, b6A, b6U) were mutated individually or in combination, were analyzed by miniDP priming assays with either of the four dNTPs. Variants are designated as uXb6Y, with X and Y defining the nt at the unpaired U and the b6 position, respectively. (**A**) Autoradiograms of individual priming assays. Note the preference of all substantially active variants for the nt complementary to the respective b6 position, except those with b6G which equally utilized dTTP and dCTP. (**B**) Graphical representation. Signal intensities were determined by phosphorimaging and related to the dGTP priming signal with wt Dε (uUb6C) which was set to 100%. Note the strong reduction in overall priming efficiency for all variants carrying a G at the unpaired U position, and the preference for utilizing the dNTP complementary to b6, except for b6G. (**C**) Schematic correlation of priming efficacy and b6 initiation site specificity with structural impact on the bulge region. RNA variants were categorized for overall priming efficacy and preference for utilizing the dNTP complementary to b6. Structural impact was assessed by the potential for new canonical plus G-U pairs. A high potential correlated with low overall priming; assessments for all variants are shown in Supplementary Fig. S5. The relaxed specificity of uUb6G and uAb6G for both dCTP and dTTP is in line with the importance of a G following the initiation site (see Fig. 6 and below). (**D**) Impact of the top bulge closing nt on priming efficacy and initiation site selection. The G-C bulge closing G-C pair in wt-Dε was swapped to c-g. Both RNAs were analyzed in parallel in miniDP priming assays with all four dNTPs. Note the drastic drop in priming efficacy but detectable maintainance of dGTP preference in the mutant. (**E**) Impact of the nt opposite the G following the bulge. Priming capacity was assessed by miniDP assays with dGTP. Note the ample options for new basepairings in mutants G-g and G-a.

Besides carrying sequence-specific determinants in its tip^32^ the stable lower stem in wt Dε expels the 5′ end of the bulge as well as the oppositely located unpaired U^26^. To test whether these features contribute to defining bulge position 6 (b6) as initiation site we deleted the unpaired U (uΔ) or replaced it by the other three nucleotides (uA, uG, uC). We then combined these mutations with all possible nt at the b6 position. The variants were designated as uXb6Y where X denotes the nt at the unpaired U position and Y that at the b6 position. Using the miniDP *in vitro* priming system we then assessed each RNA’s dNTP preference. Preferential incorporation of the dNTP complementary to b6 would indicate use of the authentic initiation site. The original priming data are compiled in Fig. 7A, and a semiquantitative evaluation in Fig. 7B.

Though complex in detail, already visual inspection of the priming signals (Fig. 7A) revealed several trends. First, the wt combination uUb6C outperformed all others, with a strong preference for dG, i.e. complementary to b6C (Fig. 7A, *upper left panel*). Replacement of the unpaired U or its deletion maintained this preference, although at lower efficacy (Fig. 7A, *upper row*). A G at b6 (uXb6G; Fig. 7A, *second row*) reduced overall priming efficiency (see below) and led, uniquely, to preferential utilization of both dC and dT when combined with the authentic unpaired U and, less pronouncedly, with an A at the unpaired U position; in all other b6G combinations signals were very low, without an evident preference for a particular nt. An A at b6 (uXb6A; Fig. 7, *third row*) caused preferential utilization of dT, especially in combination with the genuine unpaired U or an A at this position. Lastly, with a U at b6 (uXb6U; Fig. 7A, *fourth row*) dA was preferentially incorporated by all unpaired U variants, except those with a G.

The graphic representation of these data in Fig. 7B re-highlights the impact of the b6 nt on dNTP preference. In all groups but one the respective unpaired U mutants showing reasonable priming activity exerted a clear preference for the dNTP complementary to b6, confirming that this position represents the dominant initiation site. The exception was the nearly equal utilization of dCTP and dTTP by the two b6G variants with the authentic unpaired U, or an A at this position. Given the importance of a G residue following the initiation site (further confirmed below) and the presence of two consecutive Gs in the b6G variants, either G residue might take on this role (Fig. 7C), in one case specifying b6 as template for dC incorporation, in the other b5 for dT incorporation.

A common feature of the variants exerting strongly reduced priming efficacy, e.g. those carrying a G at b6 or the unpaired U position, was their potential to severely alter the bulge region architecture by new basepairings, as indicated for some representative examples in Fig. 7C and more comprehensively in Supplementary Fig. S5. In general, the absence of such stable alternative structures correlated with higher priming efficacy.

We also assessed the replication performance of all bulge region mutants (Supplementary Fig. S6). Though a thorough analysis will require further experiments, all variants except uCb6G (which had given extremely weak *in vitro* priming signals) produced detectable bands at the authentic RC-DNA and dsL-DNA positions; band intensities correlated well with *in vitro* priming efficiency. For instance, the two best-performing variants uAb6C and uCb6C (~50% and ~25% signal intensity of wt DHBV) had also given the strongest *in vitro* priming signals, with a pronounced preference for the authentic dG (Fig. 7A,B). Poor replication of the other variants is as well in line with their poor *in vitro* priming capacities, and may further be reduced in variants that, *in vitro*, preferred a nt different from dG. Hence the bulge architecture and template sequence in wt Dε are optimized for efficient synthesis of the most appropriate primer.

Altogether, these data suggested that the b6 position represents the preferred initiation site. The only (partial) shift was seen with the b6G variants (see above), underscoring the potential importance of a G residue immediately after the initiation site.

To directly address this point, we swapped the G-C pair closing the top of the bulge to C-G, while otherwise maintaining the original wt Dε sequence. This exchange drastically reduced *in vitro* priming efficiency by ~20-fold while a preference for dG utilization was preserved (Fig. 7D). In the RRL system, signals generated by the c-g mutant did not exceed those from a control without Dε RNA (not shown). Hence the G residue following the bulge sequence is indeed crucial for efficient priming from the genuine initiation site. To clarify whether the opposite C-residue (at least formally allowing formation of a “bulge-closing” G-C pair) contributes to this activity we replaced the respective C by the three other nt (bulge closing pair variants G-a, G-g, G-u). In *in vitro* dGTP miniDP priming assays both the G-g and G-a mutations massively reduced the priming signals whereas the G-u mutation was well tolerated (Fig. 7E). Though compatible with a basepairing requirement for the G residue following the initiation site, the G-g and the G-a mutations could also exert their negative impact by disturbing the genuine bulge region structure via alternative pairings, as schematically indicated in Fig. 7E.

### The presumed linear determinants for productive P protein interaction are functional in a fully unstructured upper stem context

**Figure 8:**
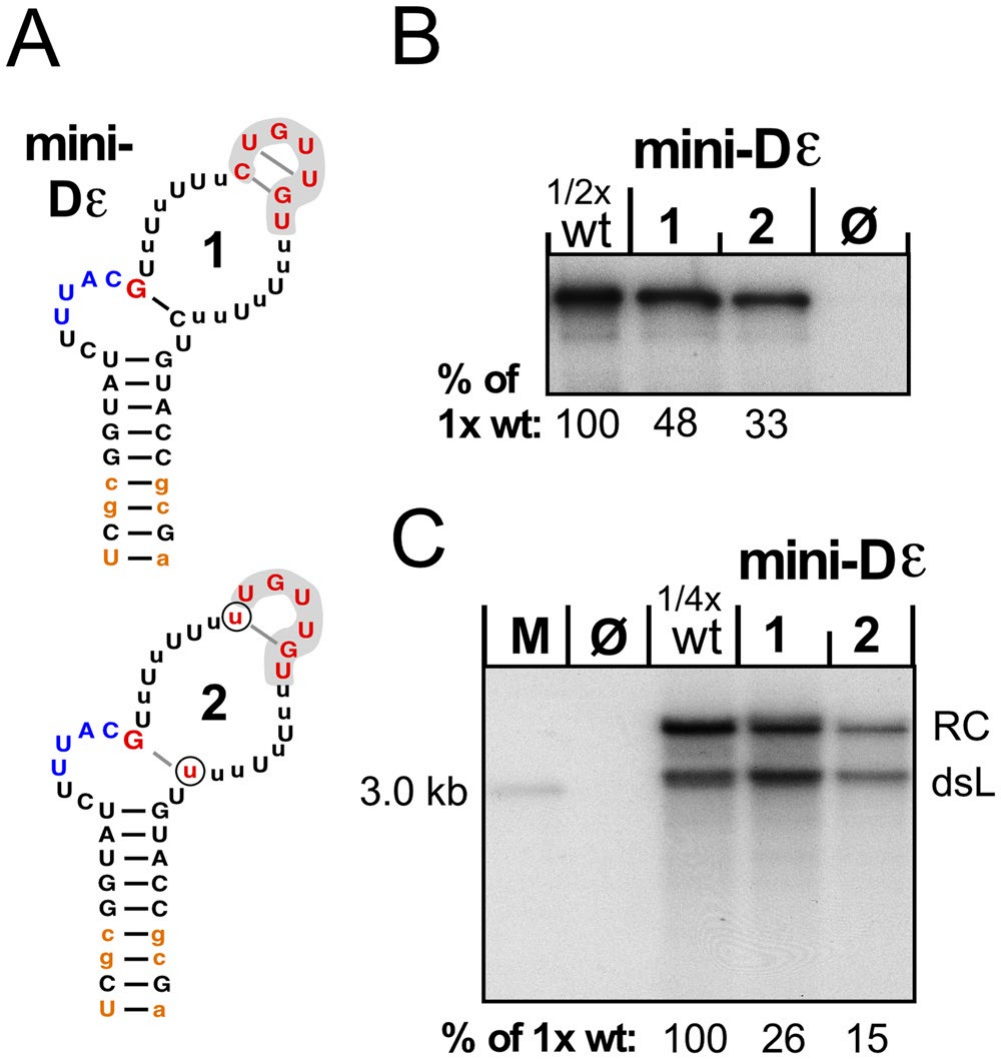
The GUUGU motif and the G following the bulge maintain functionality in the absence of a defined structure context. (**A**) Sequences of mini-Dε1 and mini-Dε2 RNAs. The two U residues distinguishing mini-Dε2 from mini-Dε1 are encircled. Lower case lettering indicates non-wt nt; those shown in orange in the lower stem were present in the RNAs used for *in vitro* priming but not in the mini-Dε pCD16_Δ3′ε vectors used to assess replication competence. (**B**) Mini-Dε *in vitro* priming activity. *In vitro* transcribed mini-Dε RNAs were subjected to miniDP priming assays with dGTP, in parallel with controls without RNA (ø) and with wt-Dε RNA; of the latter sample, only half as much of the reaction was loaded (1/2 wt). Signals were quantified by phosphorimaging and those of the variants were related to twice the intensity (set as 100%) of the 1/2 wt signal. (**C**) Mini-Dε1 and mini-Dε2 support DHBV replication. LMH cells were transfected with wt-DHBV vector pCD16_Δ3′ε or derivatives carrying mini-Dε1 and mini-Dε2 at the 5′ ε position. DNA from cytoplasmic nucleocapsids was analyzed by Southern blotting using a^32^P-labeled DHBV DNA probe.

In sum, the data described above indicated specific features of the bulge region, a G following the initiation site, plus a distant GUUGU motif as key elements for productive P protein interaction. However, all RNAs tested so far still contained additional wt-like sequence and/or structure features. As an ultimate test we therefore devised two “minimal variants” of Dε (mini-Dε1 and mini-Dε2; Fig. 8A) where these elements were presented in a completely unstructured context of runs of seven Us replacing the original upper stem. Bulge region specific features on the basal side were ensured by a stable lower stem with the authentic top five basepairs and three non-wild-type basepairs at the bottom. Mini-Dε1 maintained the original C residues opposite the G following the bulge and at loop position 1; in mini-Dε2 also these residues were replaced by U. Both RNAs were then used as templates in miniDP *in vitro* dGTP priming assays, alongside wt Dε RNA as positive control and a reaction without RNA as negative control. Remarkably, both RNAs produced very substantial priming signals approaching 50% (mini-Dε1) and 35% (mini-Dε2) of those generated by wt Dε RNA (Fig. 8B). Hence the supposed key elements are not only required but also sufficient to establish a productive interaction with P protein, without need for a specific sequence and/or structure context in the apical Dε part. Furthermore, the activity of RNA mini-Dε2 indicates that basepairing of the G residues following the initiation site and at the base of apical loop (schematically indicated in Fig. 8) is not essential because the likelihood for exactly these G-U pairs to form (as opposed to pairs involving any other of the multiple U residues) appears minute. Hence the fundamental determinants in the apical Dε part for generating a priming-active complex with P protein are of linear nature. Perhaps most surprisingly, both mini-Dε1 and mini-Dε2 supported viral replication when transfected as part of the DHBV genome in the pCD16_Δ3′ε vector into LMH cells (Fig. 8C). The presence of the mini-Dε sequences in the RC-DNAs was confirmed by direct sequencing of RC-DNA specific PCR amplicons obtained using a forward primer that binds to DHBV positions 2474–2496, i.e. upstream of the 5′ end at position 2520 of the DHBV genome in plasmid pCD16_Δ3′ε; this also excluded that the sequences were derived from transfected plasmid.

## Discussion

The hepadnaviral ε RNA element allows selective recognition of pgRNA amongst a plethora of other RNAs in a cell by P protein, activates P protein’s enzymatic function and provides the information for initiating DNA synthesis at a specific internal site. The intricate bi-partite ε stem-loop structure appears highly suited to fulfil all these requirements and the special importance of the unpaired bulge and apical loop regions, as in many other protein-RNA interactions, was experimentally proven^6, 10, 23–25, 29, 30, 33–35^. As unpaired RNA regions are defined by their flanking double-stranded regions a structural role for the connecting upper stem in maintaining and possibly orienting the bulge and loop appeared well conceivable. Moreover, the unique location of the 3′ terminal bulge nt (b6) at the junction to the upper stem double-helix could contribute to its specific use as initiation site. Not the least, the upper stem sequence could itself harbor direct determinants for a productive interaction with P protein.

The comprehensive mutational data of this study challenge all of these notions. The only fundamental determinants identifiable in the entire apical half of Dε were the GUUGU motif in the classical Dε loop sequence and a G residue following the bulge with an oppositely located pyrimidine; yet they could exert their functions in priming and initiation site selection independently of a specific RNA structure context. Hence the structural determinants in Dε are confined to the lower stem which ensures single-strandedness of the bulge and the opposite unpaired U; in addition, the specific bulge architecture must not be altered by improper basepairing.

Numerous lines of evidence support these conclusions. Varying the distance between bulge and loop had only modest effects on priming (Fig. 2B) and replication (Fig. 2D), except for the most severely altered class II mutants. *In vitro*, all variants preferred dGTP over dATP (Fig. 2B,C) and all supported formation of wt-like replicative DNA intermediates (Fig. 2D) with the same minus-strand DNA 5′ ends as in wt DNA (Supplementary Fig. S1); moreover, even two class II variants established infection *in vivo* (Supplementary Fig. S2). Hence the upper stem sequence acts at most as a flexible tether for the bulge and loop, allowing them to cooperate, within limits, at various distances from each other. Only in the most severely disabled variants −2R_II and −2LR_II (Fig. 2) may that distance be too short to allow both elements to efficiently interact with P protein.

SELEX-targeting the classical seven nt loop sequence in different contexts (Figs 4 and 5) rapidly selected the 3′ proximal wt sequence GUUGU but differing nt at the two 5′ terminal positions; these did neither substantially impair replication nor the wt-like preference for dGTP as first nt in priming. Notably, the variant loop sequence gaGUUGU was selected *in vivo*, although it lacks the ability to form a wt-like apical tetraloop (Fig. 5C). Most convincingly dispensability of a stable apical loop structure was confirmed by the priming activity and replication-competence of the mini-Dε variants (Fig. 8).

Sequence-specific features in the GUUGU motif itself were corroborated by the sensitivity of the central U residues towards mutations (Fig. 4D), extending previous data^22, 25, 28^. Hence the GUUGU motif in the classical loop sequence is a crucial *linear* determinant for Dε function.

SELEX-targeting the upper left half-stem (ULS) revealed as the only strongly selected feature a G residue immediately following the bulge, with the notable absence of G from the following four positions (Fig. 6B). Multiple mutations at the other ULS positions were compatible with replication competence (Fig. 6C) and maintained a preference for dGTP in *in vitro* priming (Fig. 6D). Hence the ULS sequence is neither crucial in itself nor is there a fundamental need for basepairing with the opposite upper right half-stem. Remarkably, variants ULS3-3 and ULS5-2 lack the potential to form more than a single G-C pair to close the bulge (Fig. 6D) which is unlikely to occur, as is particularly evident for the mini-Dε variants (Fig. 8). Hence the bulge in Dε does not have to conform to the definition of being closed on both sides, and the invariantly selected G residue following the bulge may also be considered as a linear determinant for Dε function. Its functional importance was confirmed by a drastic drop in priming efficiency upon swapping the bulge closing G-C pair to c-g (Fig. 7D).

In contrast to the virtual absence of specificity determinants in the upper stem (except the GUUGU motif and the G following the bulge), the distinct architecture of the bulge region was critical for priming activity and initiation site selection, as inferred from some low replicators from early SELEX rounds (Fig. 3B,C) and directly shown by the combinatorial mutations of the unpaired U opposite the bulge plus the b6 position (Fig. 7). These confirmed the dominant role of the b6 position as initiation site by the preference for the complementary dNTP with almost all substantially active RNAs (Fig. 7A,B); hence base-identity at b6 is not itself decisive for initiation site selection. The only exception were mutants with a G at b6 which caused equal utilization of dC and dT. Likely, in these variants both G residues at the 3′ end of the bulge can contribute to selecting the preceding residue as initiation site (Fig. 7C). Another general feature was the detrimental impact on overall priming activity of mutations allowing new stable basepairings within the bulge region (Figs 7C and S5).

Hence single-strandedness of the bulge region for at least 6 nt from the top end of the lower stem appears crucial for efficient priming, with the sixth nt predestined as initiation site; however, its efficient utilization is strongly favored by a G residue at the following position, as confirmed by the bulge closing basepair mutant c-g which still preferred dG but at a drastically lower overall priming level (Fig. 7D). Replacing the opposite C by U had only a minor impact on priming whereas G or A drastically reduced priming activity (Fig. 7E). Though suggestive of a role for a bulge-closing G-C or G-U pair, stable isolated G-C or G-U pairs are unlikely to exist, especially in the absence of supporting neighboring structures as in mini-Dε (Fig. 8). Hence a pyrimidine above the unpaired U may be part of the determinant at the top of the lower stem and/or directly contribute to initiation site selection. Alternatively, purines at this position may act inhibitory via improper intra-bulge pairings (Fig. 7E). In this and other ambiguous cases mini-Dε would provide an ideal framework for distinction, including for the question whether an A-U (or U-A) pair could functionally substitute for the G-C or G-U pair.

**Figure 9:**
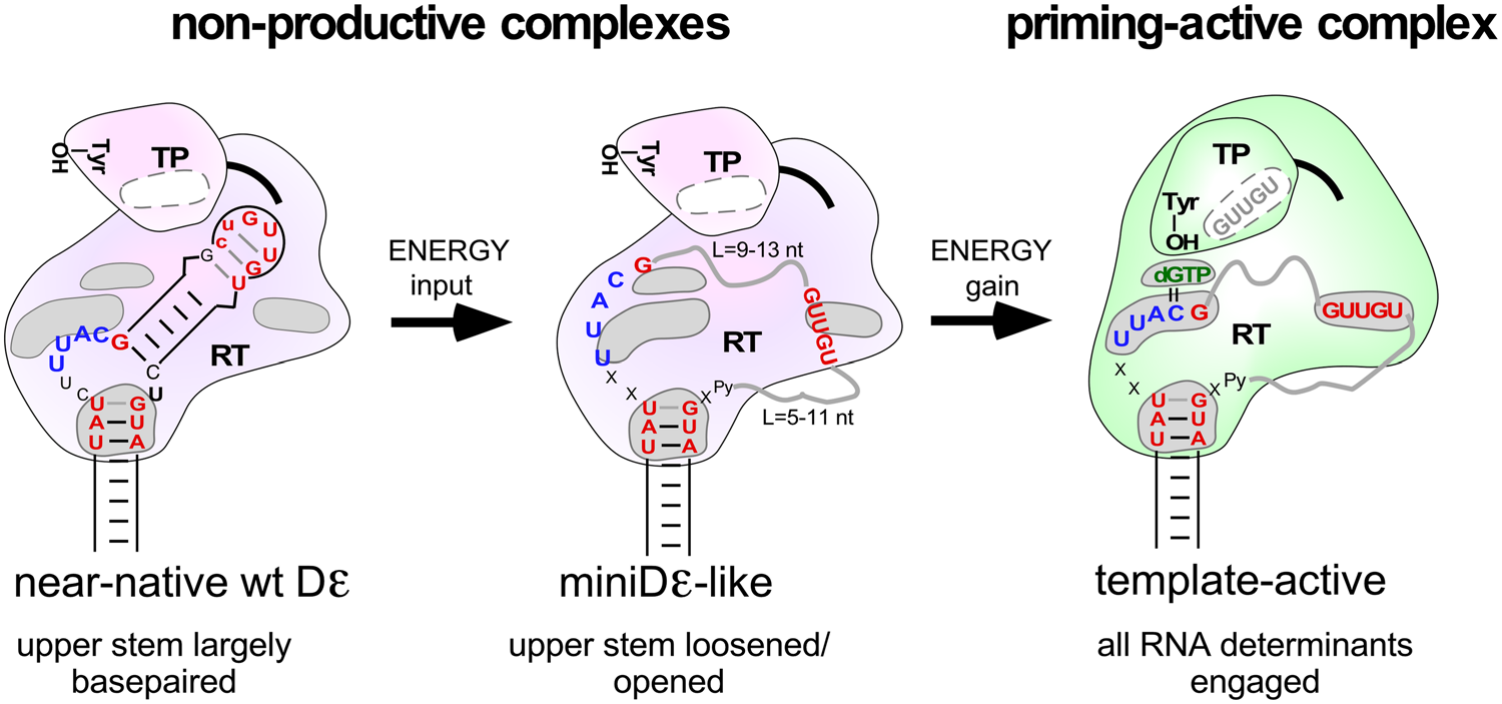
Updated model of avihepadnaviral replication initiation. The cartoons integrate key findings of this study with previous results, including the stepwise formation of a priming-active complex (Fig. 1B). The essential RNA elements in a generalized mini-Dε like sequence (center) are a stable lower stem with specific nt at its tip (red) that ensures single-strandedness of the template region (blue); this must be followed by a G (red). X denotes bulge region nt that are flexible as long as they do not interfere with the genuine bulge architecture; Py represents the pyrimidine opposite the G following the template sequence. GUUGU (red) denotes the apical loop motif, grey wavy lines the connecting sequences to the bulge region which can be of various lengths; length limits from this study are indicated. Grey areas symbolize specific binding sites on P protein; alternatively to RT, a binding site for the GUUGU motif may reside in TP (white area with a dashed outline). In the priming-active state (green) all binding sites are occupied, and the priming Tyr in TP, the incoming dGTP and the initiation site are properly aligned. Partial occupancy corresponds to non-productive binding (magenta). Whether the active state is reached depends on the energy balance between breaking existing intra-RNA interactions (higher in wt Dε than in mini-Dε) vs. the energy gained by protein binding. Mini-Dε RNA may represent a transient intermediate or a separate entry into the pathway. The order of binding site occupation and number of sites to be occupied for stable non-productive binding is not known. However, in HBV binding of the GU-rich loop motif appears as the limiting step for *in vitro* priming competence. See text for further details.

How can this new view of the Dε upper stem as a largely passive unstructured tether for the GUUGU motif (and the G following the bulge) be reconciled with the highly structured upper stem in wt Dε? A likely scenario is outlined in Fig. 9 which integrates all data sets from this study. As demonstrated by the mini-Dε RNAs, a mere three principal determinants make an RNA suitable as template for initiation site-specific protein-priming by DHBV P protein: (i) a template region followed by a G residue that is kept single-stranded by a stable stem on the basal but not necessarily the apical side; (ii) a nearby linear GUUGU motif, however without stringent distance constraints; (iii) as previously established, a stable lower stem of at least five basepairs^36^, with the top two or three carrying sequence-specific information^32^. These elements suffice to activate P protein and position the template region such that the catalytic residues of the RT domain, the dNTP binding pocket, and the last nt of the bulge (the initiation site) are all properly aligned; only then can the bond to the priming Tyr-residue in the TP domain form (Fig. 9).

Most plausibly, this is achieved by complementary sites on P protein that specifically bind the RNA determinants (grey areas in Fig. 9). One such site must exist for the 3′ proximal bulge region such that the nt at b6 can template incorporation of the first, complementary dNTP; this likely includes the crucial following G residue. A second site likely involves the nt at the top lower stem where basepairing alone is insufficient for priming activity^32^. A third binding site is predicted to accomodate the GUUGU motif or parts thereof; a strong candidate for a direct contact is the first U (L4 position) where no other nt was tolerated (Fig. 4E). Its functioning from various distances and without a rigid connection favors a role in P protein activation rather than initiation site selection. A separate loop-binding factor^28^ can be excluded because in the miniDP system only P protein, Dε RNA and buffer salts are present. The GUUGU motif may then bind within the RT domain and indirectly cause TP to accomodate a priming-active orientation in the complex; alternatively, it could bind directly to TP (Fig. 9). In all the rest of the apical Dε part, neither a specific sequence nor the ability to adopt a distinct structure are fundamentally important. To the contrary, accessibility of the few relevant RNA determinants appears as key to priming-competence.

Priming-active P protein - ε RNA complexes do not form via lock-and-key binding but in a dynamic multi-step process. For instance, various Dε RNA variants can bind to P protein but do not support priming^29, 30^, coincident with their inability to undergo the apical structural rearrangement that occurs with priming-competent RNAs^6^. Which explanation holds for which of the priming-defective variants from the current study remains to be determined. However, from the many priming-active variants we can propose that a main feature of a functional Dε upper stem sequence is to allow engagement by P protein of the specific RNA determinants at a reasonable energetic cost, defined by the energy required to break the existing intra-RNA interactions and the energy gained by the new RNA - protein interactions. Full occupancy of all RNA binding sites on P protein, likely in a stepwise process, would correlate with priming activity, and partial occupancy with non-productive binding, as also seen with HBV (see below). In this model, mini-Dε1 and mini-Dε2 represent one extreme with little or no energy input required to disrupt existing structures. Wild-type Dε with its largely basepaired upper stem would represent an intermediate case where the energy gained by productive binding to P protein is just high enough to enable the RNA rearrangement, possibly via mini-Dε-like intermediates. The tether rather than ruler function of the upper stem sequences would conform to a “fly-casting” mechanism^37^ whereby occupation of one site increases chances for engagement of nearby other RNA determinants. Further stabilizing the upper stem would increase the energetic barrier beyond a threshold and prevent *in vitro* priming^6^. In non-binding Dε variants the *a priori* accessible RNA determinants would be insufficient to sustain any stable protein interaction, in line with the dramatic influence of RNA context on protein-binding to specific RNA sequences^38^.

How relevant is this model for naturally occurring ε sequences? As the identified primordial RNA determinants are invariantly present in the ε sequences of all known avian HBVs^30^ the principal conclusions should apply to all of them. Nonetheless, there is much less natural sequence variability in the upper stem part than implicated by the *in vitro* priming activity of the mini-Dε variants. This probably reflects the multiple additional functions of the ε sequence^22^, e.g. compatibility with the mRNA function of pgRNA for core and P protein, and as part of the preC ORF from which the secreted (albeit non-esssential) e antigen is produced. Notably, all natural avian ε sequences maintain two or more canonical basepairs to close the top of the bulge and the apical loop, in seeming contrast to our data. Plausibly such local basepairing constrains the ability of the relevant ε determinants to undergo irrelevant pairings elsewhere in the pgRNA. Also, while no defined loop structure is required for the GUUGU motif to function, its presentation on a properly structured loop could still facilitate initiation of direct contacts with P protein.

The overall similarity to Dε of HBV ε (Fig. 1) suggests that key features of the model also apply to the human and other orthohepadnaviruses, however with some adaptations. The bulge in orthohepadnaviruses is not followed by G but by the sequence AAG. Possibly one of the A residues takes on the role of the G residue in Dε, or the G residue acts from a distance to the initiation site at the 3′ terminal C in the bulge^14^ and/or the following A^10, 39^. Otherwise, a specific architecture of the bulge region itself appears also crucial in HBV^13, 14, 24, 40, 41^. The classical sequence in orthohepadnaviruses is similar to that in avihepadnaviruses (CUGUGC vs. CUGUUGU) and also here the central GUG motif appears most important^24, 34^. However, any deeper mechanistic understanding will require an HBV *in vitro* priming system, ideally comprising just P protein and ε RNA; obviously, this could also serve to screen for new antivirals that interfere with protein-priming as a highly virus-specific target.

Up to date, none of the *in vitro* systems that work for DHBV has yielded authentic ε-templated protein-priming activity. Intriguingly, specific though non-productive *in vitro* binding to HBV P protein requires most of the ε RNA but not the apical loop^13, 33^, implying that failure of P protein to engage the loop sequence causes the lack of activity. One interpretation is the absence, from all systems tested, of an auxiliary loop-binding host factor^13^. Based on our model (Fig. 9) we propose instead that embedment of the motif into the highly stable upper stem structure in HBV ε precludes a productive, direct interaction with P protein *in vitro*. Hence destabilizing the upper stem should lower the energy barrier that prevents rearrangement of the HBV RNA into a new, productive conformation. If the upper stem in HBV ε harbors more specific information than in Dε, as suggested by earlier studies^13, 28, 41^ and its near universal sequence conservation in the mammalian viruses, the in-cell SELEX methods developed here lend themselves to identifying mutants that combine decreased upper stem stability with replication- and consequently priming-competence.

## Materials and Methods

A detailed description of the in-cell SELEX procedures is provided in Supplementary Methods.

### Plasmid constructs

The parental DHBV16 expression vector pCD16 carries a 1.1 × DHBV16 genome (GenBank accession no. K01834; DHBV16 positions 2520–3021/1–2816) under control of the cytomegalovirus (CMV) immediate-early (IE) enhancer promoter. In its derivative pCD16_Δ3′ε the 3′ copy of Dε was made non-functional by a 40 nt deletion (DHBV16 positions 2568–2607). For *in vitro* transcription, pUC19T7 vectors were used which carry the Dε sequence, or derivatives thereof, under control of the bacteriophage T7 RNA polymerase promoter^22, 30^. Vectors encoding the modified Dε sequences S1 and S12 have previously been described^22, 30^. Additional mutations were introduced via conventional cloning of PCR products obtained using mutagenic primers, or via the Q5 mutagenesis kit (NEB). Generation of the SELEX vector pools is detailed in Supplementary Methods. All construct were verified by Sanger sequencing.

### *In vitro* priming

*In vitro* Dε transcripts were generated from the respective pUC19T7 vectors linearized immediately after the Dε cassette^22, 30^ using the T7 MEGAScript kit (Ambion). Priming assays were performed using either DHBV P protein *in vitro* translated in rabbit reticulocyte lysate^6^, or using bacterially expressed DHBV miniDP protein (which does not require chaperones for activation) and 1 µM *in vitro* transcribed Dε RNA plus the desired α^32^P-labeled dNTP (at equal specific activity when comparing different dNTPs) as previously described^31^; Covalently ^32^P labeled P protein resulting from successful protein-priming was detected by autoradiography and/or phosphorimaging (Typhoon system, GE Healthcare) after SDS-PAGE separation. Band intensities were quantified by phosphorimaging, using ImageQuant software.

### Cell culture and transfection

Chicken LMH hepatoma cells were cultured and transfected using Mirus TransIT-LT1 reagent (Mirus) as previously described^22^.

### Detection of viral gene products and nucleic acids

Detection of cytoplasmic capsids by immunoblotting after native agarose gel electrophoresis (NAGE), isolation of viral nucleic acids associated with cytoplasmic nucleocapsids and extracellular viral particles after enrichment by polyethylen glycol precipitation, and Southern blotting using a^32^P labeled DHBV DNA probe were all conducted as previously described^22^. The presence of the mini-Dε1 and mini-Dε2 sequences in nucleocapsid-associated viral DNAs from LMH cells transfected with the respective pCD16_Δ3′ε plasmid derivatives was verified by sequencing of PCR amplicons obtained using a forward primer matching DHBV positions 2474–2496 and and a reverse primer complementary to positions 2821–2844.

### *In vivo* infection of ducklings

All animal experiments were approved by the Regierungspräsidium Freiburg (project G02/36) and performed in compliance with German animal welfare legislation at a registered facility of the University Hospital Freiburg under veterinary supervision. Two- to three-day-old Pekin ducklings were inoculated with a dose of 10^8^ vge of transfection-derived virus and viremia over time was monitored using a one-step qPCR with a lower detection limit of about 10^5^ vge/ml^22, 42^. For sequencing of the Dε regions, primers DR1-SpeI DHBV+ (aaaaaaactagTACACCCCTCTCCTTCGGAGC; the non-DHBV 5′ sequence in lower case letters creates a SpeI restriction site) and D2738- (TTAGCATCTCTAACAAGATCATC) spanning DHBV positions 2537 to 2738 were used^22^.

### In-cell and *in vivo* SELEX procedure

A detailed account of the generation of the SELEX vector pools and the selection procedure, including the built-in precautions to minimize contamination with wt-DHBV sequences during the multiple cloning and amplification steps as well as a functional validation is given in Supplementary Methods. In brief, a ~160 bp Dε comprising DNA fragment was created via PCR using one synthetic oligonucleotide carrying the desired randomized region (the 6 nt in the bulge, the 7 nt of the classical loop, or the 7 5′ proximal nt of the upper left half-stem) as template, plus two oligos acting as forward and reverse amplification primers. The product was extended in a second PCR to generate a ~2 kb DHBV fragment which was finally cloned into a special recipient pCD16_Δ3′ε derivative, carrying a nonrelated 1.2 kb stuffer DNA instead of the 5′ proximal DHBV sequence. For the in-cell SELEX, the vector DNA pooled from ten- to twenty-thousand individual colonies was transfected into LMH cells. Three days post transfection, DNA associated with viral particles (secreted enveloped virions and in some cases intracellular nucleocapsids, as indicated) was used as template for PCR amplification of a ~2 kb genome segment harboring the Dε region from which a new vector pool was reconstituted. This procedure was repeated from five to thirteen times. Selection progress was monitored by sequencing the resulting new vector pools plus various individual plasmid clones from each round. Functionality of the pools and of select clones with known sequence was assessed by Southern blotting, using viral DNA from cells transfected with pCD16 and/or pCD16_Δ3′ε as reference. For *in vivo* selection, two ducklings were inoculated as described above with virions from a first round transfection-derived vector pool carrying a randomized loop sequence in the context of variant S1^22^. Sequence analyses were performed on PCR products obtained using DNA from serum-borne virions as template at the indicated time points post inoculation as described^22^.

### Data Availability

All data generated or analysed during this study are included in this published article and its Supplementary Information files.

## Electronic supplementary material

Supplementary Information
