# Supplementary material for: Few basepairing-independent motifs in the apical half of the avian HBV ε RNA stem-loop determine site-specific initiation of protein-priming
Source: Sci Rep. 2017 Aug 2;7:7120. doi: 10.1038/s41598-017-07657-z (PMC5541001; doi:10.1038/s41598-017-07657-z)
Supplement: Supplementary file 1 — Supplementary Information [file 41598_2017_7657_MOESM1_ESM.pdf]

# Few basepairing-independent motifs in the apical half of the avian HBV ε RNA stem-loop determine site-specific initiation of protein-priming

**Markus Gajer[§1], Katharina Dörnback[§1], Christine Rösler[§1], Bernadette Schmid[1], Jürgen Beck[§*1], Michael Nassal[*1]**

[1]University Hospital Freiburg, Department of Internal Medicine II / Molecular Biology; Hugstetter Str. 55, D-79106 Freiburg, Germany

[§]These authors contributed equally

*Corresponding authors

## SUPPLEMENTARY INFORMATION

**Section 1: Supplementary Figures S1 - S6**

**Section 2: Supplementary Methods - In-cell SELEX procedure**

# SECTION 1: SUPPLEMENTARY FIGURES S1 - S6

## *Gajer et al.* Supplementary Figure S1

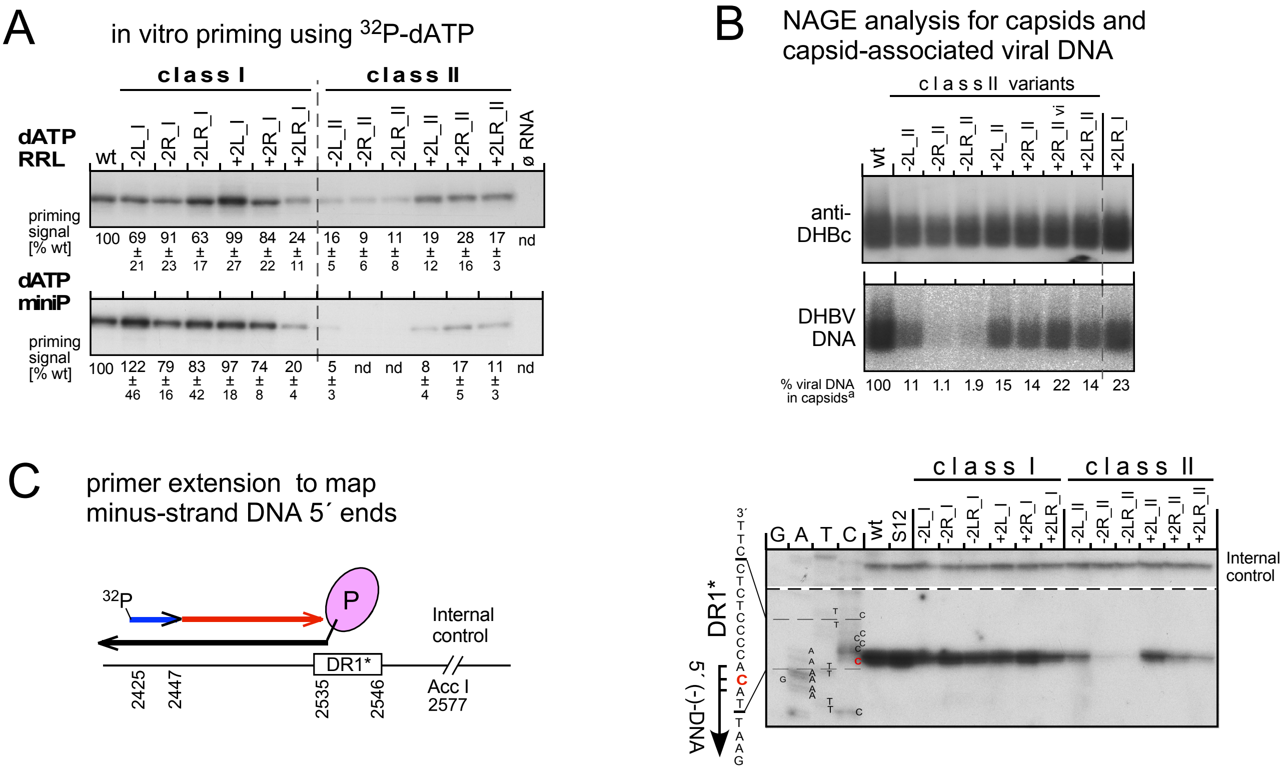


**Supplementary Fig. S1. Functional impact of varying the distance between Dε bulge and apical stem. (A) In vitro priming with $\alpha^{32}$P-dATP.** Priming assays were performed in RRL or in the miniP system as in Fig. 2B. Priming signals were markedly reduced compared to dGTP priming (see Fig. 2C for a direct comparison) but the relative signal intensities between wt Dε and all variants were maintained; hence neither class I nor class II modifications had a major impact on dNTP preference. Numbers on the bottom of each autoradiogram show the priming signal intensities ± SD (n=4) relative to the signal produced by wt Dε RNA as template; nd, not detectable. **(B) Viral DNA content of capsids.** Cytoplasmic lysates of transfected LMH cells were analyzed by NAGE for DHBV capsids by immunoblot with monoclonal antibody 2B9-4F8 (Vorreiter et al. 2007), or in parallel by molecular hybridization with a $^{32}$P-labeled DHBV probe. DNA contents per capsid (numbers at the bottom) were calculated by relating the DNA signal intensity (by phosphorimaging) to that of the respective immunoblot signal (by densitometric scanning), with the ratio from the wt Dε sample set to 100%. **(C) Approximate mapping of minus-strand DNA 5´ ends.** DNAs from cytoplasmic nucleocapsids, spiked with pCD16 plasmid DNA linearized at the Acc I at position 2577 of DHBV (internal control), served as template for primer extension using a (+)-polarity 5´ $^{32}$P labeled oligonucleotide corresponding to DHBV16 coordinates 2425-2447 and Vent polymerase (Maguire and Loeb, 2010). Products were separated on a 10% polyacrylamide / 7 M urea gel alongside a sequencing ladder produced on pCD16 using the same primer. While signal intensities showed a very similar dependence on the specific Dε mutations as observed in capsid DNA content (Fig. S1B) and DNA replicative intermediates revealed by Southern blotting (Fig. 2D), the main primer extension products from all variants migrated to virtually the same positions (± 2 nt) as those from wt DHBV. Their mapping to just below the C4 cluster in DR1* (identifiable despite the limited quality of the sequencing ladder) is in line with the published major (-)-DNA start around position 2536 (Maguire and Loeb, 2010). Hence even massive length variations of the Dε upper stem still allowed generation of P protein-linked primers capable of translocation to the proper acceptor site, suggesting that despite strongly reduced priming efficiency important features of initiation site specificity are preserved.

**Supplementary references:**


Vorreiter J, Leifer I, Rösler C, Jackevica L, Pumpens P, Nassal M. 2007. Monoclonal antibodies providing topological information on the duck hepatitis B virus core protein and avihepadnaviral nucleocapsid structure. J. Virol. 81(23):13230-4

Maguire ML, Loeb DD. 2010. cis-Acting sequences that contribute to synthesis of minus-strand DNA are not conserved between hepadnaviruses. J. Virol. 84(24):12824-31

# *Gajer et al.* Supplementary Figure S2

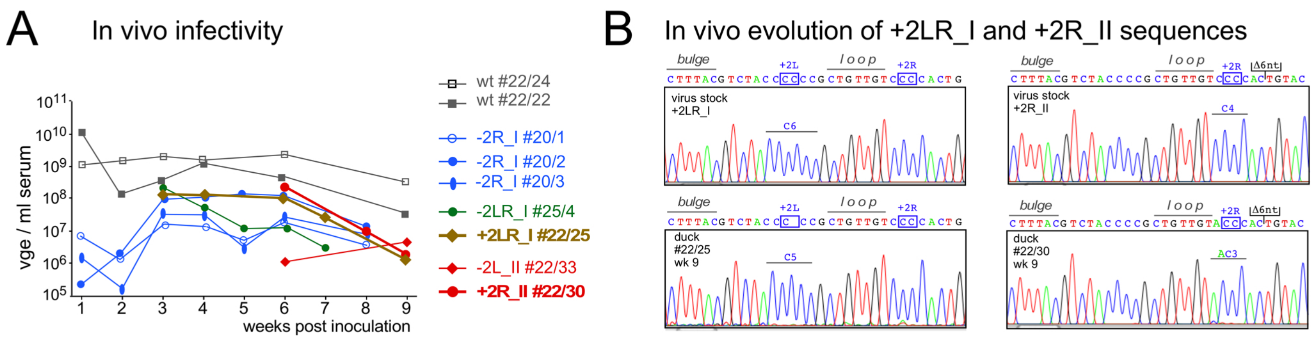


**Supplementary Fig. S2. DHBVs carrying class I and class II mutations in Dε can establish infection in ducks.** Three two-day old ducklings each were inoculated with viral particles from LMH cells transfected with the indicated constructs, and serum samples were collected weekly until week 8 or 9 post inoculation as previously described (Schmid et al. 2011). **(A) Viremia.** Viremia (as viral genome equivalents per ml [vge/ml]) was determined by a non-nested qPCR with a lower limit of detection (LLD) of ~100 genome copies per sample or 10E5 copies per ml serum (Dallmeier et al. 2007). Only data from animals giving signals above the LLD are shown. As in previous experiments (Schmid et al. 2011) the two wt-DHBV infected ducks (#22/24 and #22/22) presented with very high titers already at the first time point. In comparison, all mutants showed a delayed onset of the highly viremic phase, and maximal titers remained 2-3 log lower. **(B) Sequencing confirms descendance of low-titer late onset viremias from the mutant inocula.** To exclude that the viremias had arisen from low level contamination with wt-DHBV, PCR-amplified Dε -comprising genome segments derived from the inocula vs. the week 8 or 9 serum DNA were compared by direct sequencing. For all variants the inocula produced the expected sequences, with no signs of contamination, and the same sequences were found in the circulating virion DNAs (not shown), except for animals #22/25 (+2LR_I) and #22/30 (+2R_II); here the serum samples contained the key signatures of their parental inocula but also one mutation each. Serum virus from the recombinant +2LR_I stock had lost one out of the 6 Cs in a row preceding the loop (*left two chromatograms*); in serum virus from the +2R_II stock the C4 motif following the loop was changed to ACCC while the class II-specific 6 nt deletion was still present (*right two chromatograms*). As the respective mutants have never been produced in the lab, their presence in serum strongly supports their descendance from the recombinant stocks by limited evolution (Schmid et al. 2011).

**Supplementary references:**

Dallmeier K, Schultz U, Nassal M. 2007. Heterologous replacement of the supposed host determining region of avihepadnaviruses: high in vivo infectivity despite low infectivity for hepatocytes. PLOS Pathog. **4**, e1000230

Schmid B, Rösler C, Nassal M. 2011. A high level of mutation tolerance in the multifunctional sequence encoding the RNA encapsidation signal of an avian hepatitis B virus and slow evolution rate revealed by in vivo infection. J. Virol. 85(18):9300-13

# *Gajer et al.* Supplementary Figure S3

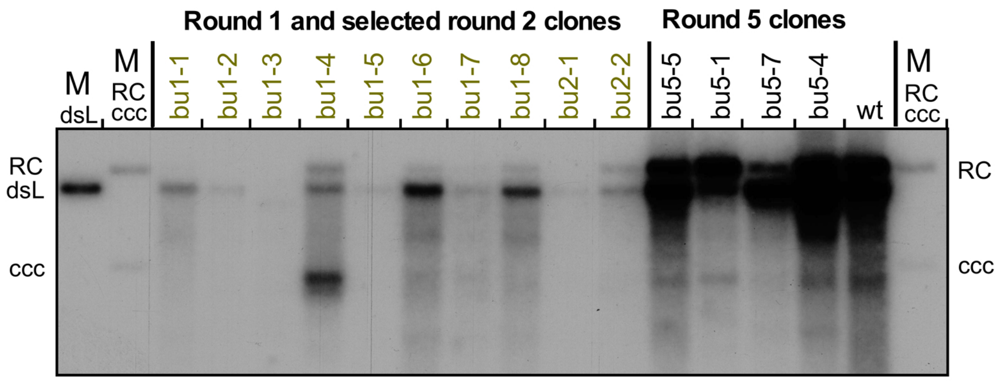


***low replicators from round 1 and 2:***

```
wt......GGTAT C T T T A C GTCTA
bu1-1...GGTAT a a T a A g GTCTA
bu1-2...GGTAT t T a g g g GTCTA
bu1-3...GGTAT C a g g A a GTCTA
bu1-4...GGTAT t a T a t a GTCTA
bu1-5...GGTAT t a g a g g GTCTA
bu1-6...GGTAT C a a T t t GTCTA
bu1-7...GGTAT C c T a t C GTCTA
bu1-8...GGTAT t g a T t t GTCTA
bu2-1...GGTAT a a g a g g GTCTA
bu2-2...GGTAT C c a T t C GTCTA
```

***non-wt-bulge high replicators from round 4 and 5***

```
wt......GGTAT C T T T A C GTCTA
bu5-5...GGTAT C a T T A C GTCTA
bu5-1...GGTAT t a T T A C GTCTA
bu5-7...GGTAT a T T T A C GTCTA
bu5-4...GGTAT C T T T A C GTCTA
```

**Supplementary Figure S3. Rapid in-cell selection from the bulge pool of well-replicating Dε sequences carrying a wild-type template sequence.** To validate the in-cell SELEX procedure eight randomly picked clones from round 1 (bu1-1 to bu1-8) were sequenced and transfected alongside two clones with highly divergent bulge sequences from round 2 (bu2-1, bu2-2), and four round 5 clones diverging from the wt-bulge sequence at two positions (bu5-1), one position (bu5-5, bu5-7) or not at all (bu5-4; see also Fig. 3C) plus the wt-DHBV vector pCD-Δ3´ε (wt) into LMH cells; then intracellular nucleocapsid-borne DNAs were analyzed in parallel by Southern blotting; M dsL and M RC,ccc are markers for the positions of double-strand linear (dsL), relaxed circular (RC) and covalently closed circular (ccc) DHBV DNA. As shown in the autoradiogram, a high divergence from the wt-bulge sequence in the early round clones correlated with reduced but not absent replication competence. Conversely, all round 5 clones replicated at wt-like levels and carried the genuine template sequence TTAC; however, the two preceding bulge positions exerted some tolerance towards mutation. The reasons for the overall low replication performance of the round 1 clones and the in part divergent DNA patterns (e.g. clone bu1-4) and/or ratios of RC-DNA to dsL DNA have not been examined but may relate to formation of improper bulge architectures (see Fig. 3D) and/or transfer of the non-wt primers to improper 3´ acceptor sites. Together, these data strongly supported the suitability of the in-cell SELEX procedure to select replication-competent Dε sequences.

*Gajer et al.* Supplementary Figure S4

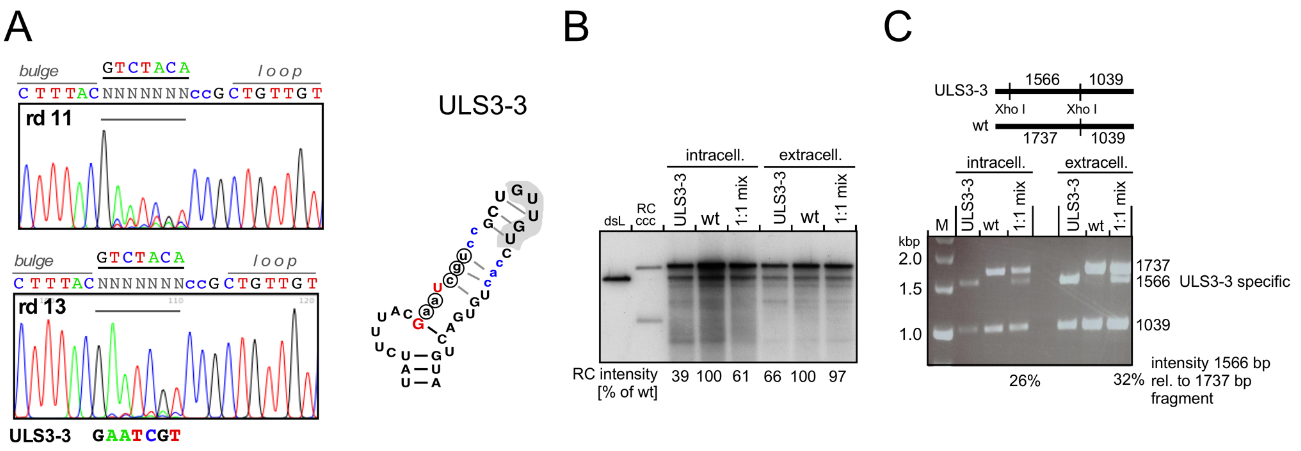


**Supplementary Fig. S4. Slow selection from the ULS pool of the winning sequence ULS3-3 and its functional characterization.** Different from the bulge and loop pools no selection towards a preferred sequence was seen for the ULS pool until round 6, except for the G residue immediately following the bulge (Fig. 6B); many different individual sequences from early rounds were principally replication-competent (Fig. 6C). Only after 13 rounds did clone ULS3-3 emerge as winning sequence. **(A) Pool sequence chromatograms after round 11 and round 13.** Some complexity in the ULS pool persisted until round 11, but an enrichment of ULS3-3 became evident; after round 13, clone ULS3-3 was predominant. In the structure scheme, non-wt nt in the parental variant S12 are shown in blue lower case; in the randomized ULS segment wt-nt are indicated in red capitals and non-wt nt in the selecteed variant ULS3-3 as encircled lower case letters. **(B,C) Functional comparison of wt-DHBV vs. clone ULS3-3.** LMH cells were transfected with wt pCD16, clone ULS3-3, or a 1:1 mixture of both plasmids. **(B) Southern blot of viral DNAs in intracellular and extracellular particles.** Signal intensities for RC-DNA were quantified by phosphorimaging and those from the wt vector were set as 100%; relative signal intensities of the other samples are indicated at the bottom. Accordingly, ULS3-3 produced 40-60% as much RC-DNA as wt DHBV. **(C) Proportion of ULS3-3 DNA in progeny DNA after cotransfection with wt DHBV.** 2.8 kb PCR fragments derived from the viral DNA preparations in (B) were incubated with Xho I which has a single recognition site in wt DNA but a second recognition site in ULS3-3 DNA; this causes the wt-specific 1737 bp fragment to be cut into a 1566 bp plus a 171 bp fragment. Digested DNAs were separated by agarose gel electrophoresis and stained by ethidiumbromide. Quantitation of the 1737 bp vs. the 1566 bp bands from the mixed transfection samples indicated the presence of about one third ULS3-3 DNA.

*Gajer et al.* Supplementary Figure S5

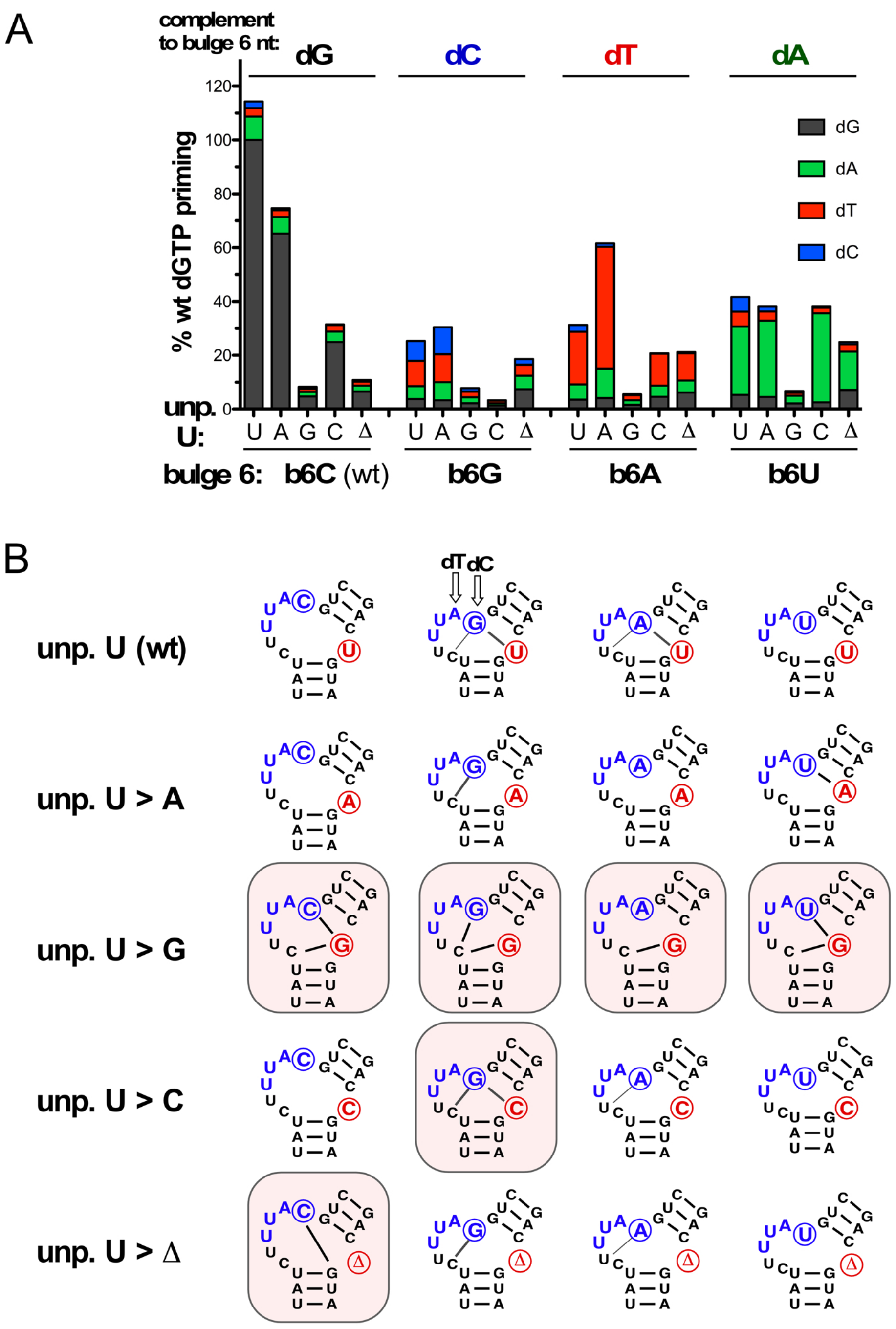


**Supplementary Fig. S5. Negative impact on in vitro priming efficiency but not initiation site selection by stable alterations of the genuine bulge architecture. (A) Overall in vitro priming efficacies and dNTP preferences of bulge region mutants.** The graph is identical to that in Fig. 7B. **(B) Hypothetical secondary structure schemes of the mutants´ bulge regions.** Target sites at bulge position 6 (b6) and the unpaired U residue are encircled. The schemes assume a similar overall base-pairing pattern as in the bulge region of wt Dε (upper left structure) and account only for the potential of the mutations at b6 and the unpaired U to locally modify the bulge architecture by introducing new basepairs. Most deleterious for priming were stable non-wt-like structures which shorten the original bulge size by extending existing double-helices (i.e. the lower or upper stem) by new G-C or C-G pairs (*highlighted by pink boxes*). For instance, all variants with a G at the unpaired U position could sequester the C at the b1 into a base-pair that extends the lower stem; a G at b6 combined with an "unpaired" C would extend the upper stem; and a C at bulge position 6 combined with deletion of the unpaired U could pair with the G on the right top of the lower stem, replacing the weaker, genuine U-G pair. Priming signals of variants containing these features were so weak that a clear preference for a specific dNTPs was difficult if not impossible to determine. Hence the distinct architecture of the authentic Dε bulge region with six unpaired residues and the unpaired U on the opposite side appears to importantly contribute to priming efficiency and proper initiation site selection.

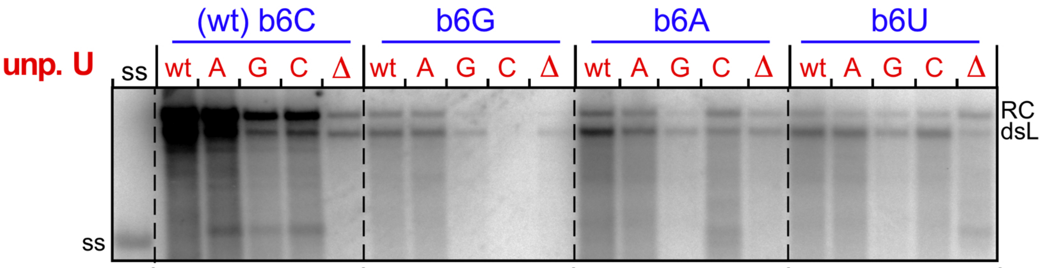


| | (wt) b6C | | | | | b6G | | | | | b6A | | | | | b6U | | | | |
|---|---|---|---|---|---|---|---|---|---|---|---|---|---|---|---|---|---|---|---|---|
| unp. U | wt | A | G | C | Δ | wt | A | G | C | Δ | wt | A | G | C | Δ | wt | A | G | C | Δ |
| % FL-DNA signal ± SD | 100 | 54 ± 20 | 16 ± 11 | 26 ± 6 | 3.9 ± 2.8 | 6.2 ± 2.8 | 4.7 ± 0.3 | 3.1 ± 0.9 | 0.2 ± 0.1 | 0.5 ± 0.3 | 16 ± 7 | 10 ± 3 | 3.0 ± 1.8 | 5.7 ± 1.5 | 2.4 ± 1.3 | 6.5 ± 3.3 | 8.4 ± 3.4 | 2.7 ± 1.3 | 5.5 ± 3.3 | 2.9 ± 1.9 |
| in vitro priming [% of wt/dG]: dG | **100** | **65** | 4.7 | **25** | 6.5 | 3.7 | 3.3 | 2.2 | 1.3 | 7.4 | 3.5 | 4.1 | 1.6 | 4.6 | 6.2 | 5.3 | 4.5 | 2.1 | 2.5 | 7.1 |
| dA | 8.7 | 6.2 | 1.7 | 3.9 | 2.2 | 4.8 | 6.8 | 2.2 | 0.9 | 5.0 | 5.7 | 11 | 1.7 | 4.1 | 4.5 | **25** | **28** | 2.9 | **33** | 14 |
| dT | 3.2 | 2.5 | 1.2 | 2.4 | 1.6 | **9.4** | **10** | 2.1 | 0.9 | 4.1 | **20** | **45** | 1.9 | **12** | **10** | 5.5 | 3.5 | 1.1 | 2.1 | 2.7 |
| dC | 2.4 | 0.8 | 0.7 | 0.2 | 0.6 | **7.4** | **10** | 1.3 | 0.2 | 2.1 | 2.5 | 1.3 | 0.3 | 0.2 | 0.4 | 5.4 | 1.8 | 0.6 | 0.4 | 0.9 |

**Supplementary Fig. S6. Similarly negative impact of bulge architecture alterations on in vitro priming and replication competence.** pCD-Δ3´ε vectors harboring the indicated bulge position 6 (b6) and unpaired U mutations were transfected into LMH cells, and intracellular nucleocapsid-borne DNAs were analyzed by Southern blotting. Signal intensities for full-length viral DNAs (i.e. RC-DNA + dsL DNA) were quantified by phosphorimaging and are indicated ± SD (n=3) as percent of the signal obtained with the wt vector which was set to 100%. The table below shows each variants´ relative in vitro priming activity with a specific dNTP (Fig. 7); usually the dNTP complementary to the b6 position had given the strongest signals (*highlighted in red bold face*), except for some of the b6G variants which utilized dCTP and dTTP nearly equally well. Although replicative DNA formation depends on various additional parameters including pgRNA encapsidation and production of a primer that can efficiently translocate to the 3´ acceptor (Fig. 1) there was a good correlation between in vitro priming and replication competence for most variants; for instance, variant uAb6C with the highest dGTP in vitro priming activity (65% of wt Dε) also replicated to the highest levels (~50% of wt DHBV); conversely, variant uCb6G had given only marginal priming signals with any dNTP and also yielded the weakest of all replicative DNA signals. Hence priming per se appears as one of the most decisive steps in replication, and at least for the DHBV mutants analyzed here is not overruled by the numerous factors present in intact cells.

# SECTION 2: Supplementary Methods

## In-cell SELEX procedure

### General outline

The procedure relies on transfection of a pool of DHBV expression vectors carrying randomized target regions, here within the Dε sequence, into LMH cells. Only functional Dε sequences are replication-competent and yield viral progeny genomes (replication-dependent selection). These are PCR amplified in vitro to yield a new vector pool enriched for functional Dε sequences; repeated application of the procedure is expected to select the best-performing individuals. As outlined below, various precautions were taken to minimize contamination of the pools with wt DHBV.

### 1. Generation of DHBV vector pools with region-specifically randomized Dε sequence

As outlined in Figure (a), in a first PCR (25 cyles, 50°C annealing temperature) using Pfx polymerase (Invitrogen), synthetic (+)-polarity 72-mer oligos carrying mixtures of all four nt at the desired positions (DepsRandBulge+; DepsWTRandLoop+; DepsS1RandLoop+; DepsS12RandULS+) were amplified using forward primers DepsSelex(+)Sal and DepsRandBulge(+)Sal, respectively, which provide a 5´proximal Sal I site (matching the Sal I site in the pCD16 vector series preceding the 5´ nt 2520 of the cloned DHBV16 genome) plus the 68-mer reverse primer Deps(-) SelexBsmB1 which introduces via two silent mutations (DHBV pos. 2661 and 2665) a Bsm BI site [CGTCTC] at core protein codons A5, S6 and R7 (GCT TCT AGA GCC to GCg T*CT cGA* GCC; mutations in lower case, Bsm BI site underlined); this also changes the genuine Xba I site at pos. 2662 (red) into an Xho I site (red italics) as genetic marker; for orientation, the core gene start is also indicated.

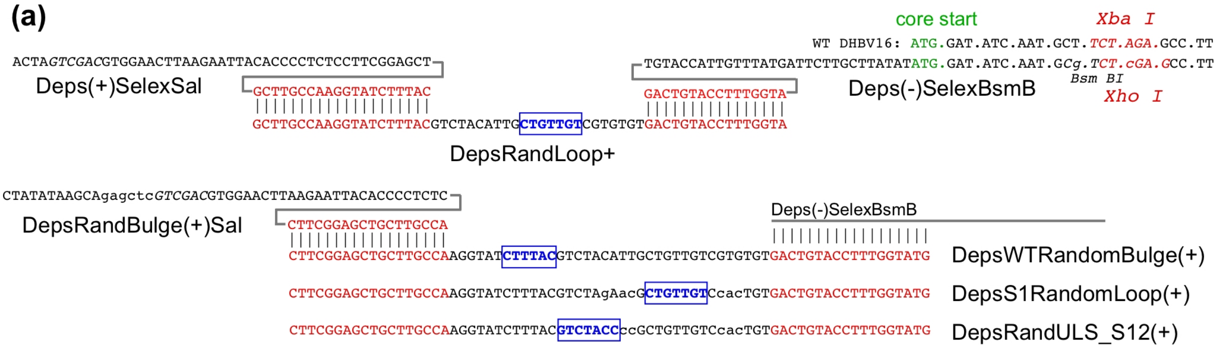


**Supplementary Methods Figure (a). Scheme for generation of PCR products with randomized Dε regions.**

The PCR products from (**a**) served as forward megaprimers in a second PCR using DHBV reverse primer D26830- (DHBV16 nt 2235-2255) and plasmid pCD16-Deps68 as template (Supplementary Methods Figure b; 2nd PCR) which carries the truncated Dε sequence -2LR_II at both ends, excluding inadvertent formation of wt Dε in the resulting 2,780 bp PCR products. These were digested with Sal I and Bst XI (corresponding to the unique Bst XI site at pos. 1433 in DHBV16) and the 1.9 kb Sal I - Bst XI fragments were cloned into recipient vector pCD16-SalBsm-stuffer-Δ3´ε (**b**). This plasmid carries 1.2 kb of stuffer DNA between the 5´ Sal I site and DHBV nt 2661, and thus lacks 5´ Dε; in addition, 40 nt of the 3´ Dε copy are deleted (**b**). Thanks to the stuffer DNA the desired 4.3 kb Sal I - Bst X1 recipient fragment for the PCR products is easily purified from incompletely digested vector. Residual parental vector does not yield intact DHBV. The non-palindromic Bst XI 5-base overhang can only unidirectionally ligate. After ligation and transformation of 1/20 ligation reaction into Top10 E. coli cells we routinely obtained 1,500-2,000 colonies per 10 cm Petri dish; control ligations of only the vector fragment gave <100 colonies. The colonies from 10 dishes each were washed off the plates, combined and regrown in 200 ml LB medium for 3 h. Plasmid DNA was isolated using a commercial kit (Qiagen), yielding the starting (round ø) pCD16-Δ3´ε vector pools; these were transfected into LMH cells as described in Materials and Methods.

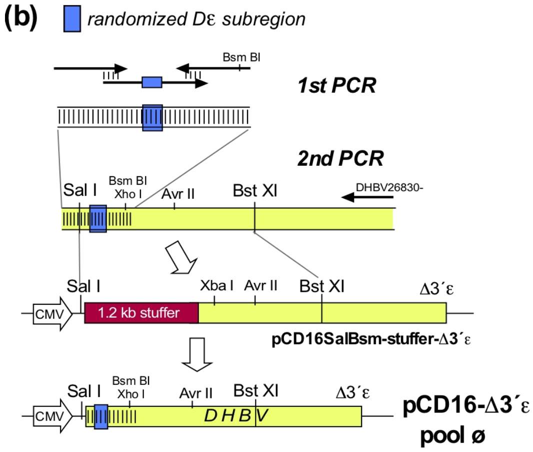


**Supplementary Methods Figure (b). Scheme for generation of DHBV expression vector pools with randomized Dε regions.** See text for details.

### 2. Selection, reamplification of the Dε region from viral DNA and production of the next generation of DHBV expression vector pools

As shown in Supplementary Methods Figure (c), four days post transfection of the plasmid pools viral progeny DNA was isolated from cytoplasmic lysates, and from extracellular virions as described in Materials and Methods. Equal aliquots of each preparation were analyzed for replicative DNAs by Southern blotting. As the amounts of virion DNA were usually low after the early selection rounds, the more abundant intracellular viral DNA was used as template for in vitro amplification; lateron, virion-derived DNA was used. The 48-mer oligo DepsSelex(+)ext matching DHBV16 nt 2520-2544 and providing a 5´ extension matching the pCD16 vector sequence upstream of the Sal I site served as forward primer, and oligo D26830- as reverse primer. The PCR products were digested with Sal I and Avr II (DHBV position 433) and the desired 906 bp fragment was cloned between the Sal I and Avr II sites in the stuffer vector. Transformation, plasmid pool isolation and sequence characterization were performed as with the starting plasmid pools. The new plasmid pools were again transfected into LMH cells, and the whole procedure was repeated for the desired number of selection rounds.

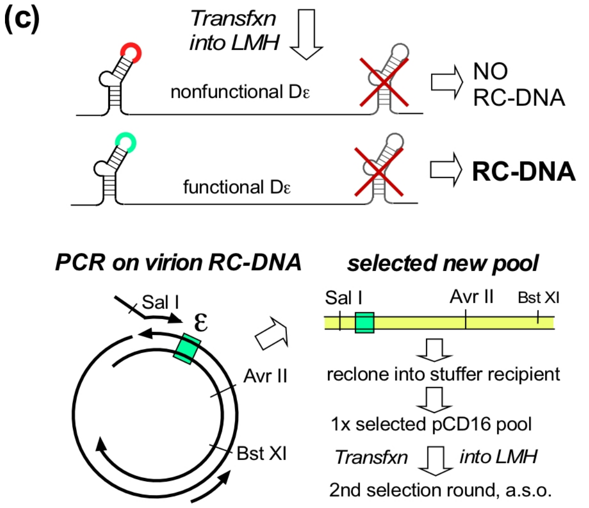


**Supplementary Methods Figure (c). Schemes for selection of functional Dε sequences and generation of progeny vector pools.** *Top:* Transfection of the pCD16-Δ3´ε pools produces pgRNAs lacking 3´ Dε and carrying non-functional (red) or functional (green) 5´ Dε. Only the latter pgRNAs will yield RC-DNA. *Bottom:* In vitro amplification and cloning scheme for progeny vector pools enriched for functional 5´ Dε sequences.

### 3. Confirmation of positive selection for replication-competence

To confirm the expected selection of replication-competent Dε sequences from the vector pools, LMH cells were transfected with wt pCD16, its derivative pCD16-Δ3´ε with a 40 bp deletion in the 3´ Dε copy as present in the vector pools, the stuffer vector from (b), or the unselected round ø vector pool (here from the loop pool). Next nuclease-resistant DNA from cytoplasmic lysates was analyzed by Southern blotting using a $^{32}$P-labeled DHBV probe (*left panel* in Supplementary Methods Figure d). pCD16-Δ3´ε yielded equivalent amounts of replicative DNAs as pCD16, confirming the absence of a negative impact of the deletion in 3´ε. The stuffer plasmid gave no detectable signals, and the round ø pool gave very weak signals, in line with a low frequency of functional sequences. The *right panel* in (d) shows a Southern blot for viral DNAs from intracellular and extracellular particles from cells transfected with pools obtained after 2, 3, and 5 selection rounds. The increasing signals indicate an increasing fraction of well replicating sequences, confirmed by decreasing pool complexity and enrichment of individual sequences seen by pool sequencing and sequencing of individual clones.

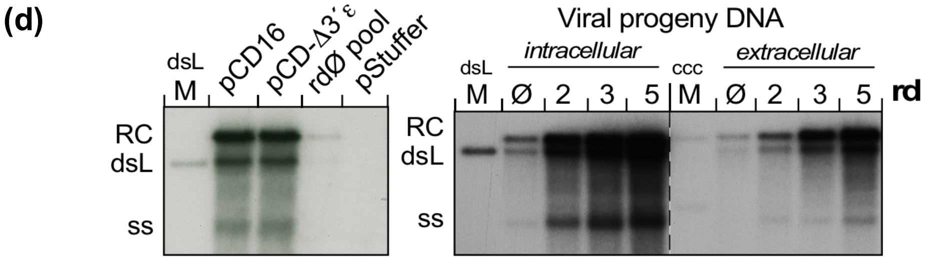


**Supplementary Methods Figure (d). Southern blotting confirms enrichment of functional Dε sequences.** See text for details.

Similar results were obtained with the other vector pools, except the upper left half-stem (ULS) pool which yielded relatively strong DNA signals early-on but with an only slow increase per selection round, in line with a slow decrease in pool complexity (Fig. 6 and Supplementary Fig. S4 online).
